# Learning capabilities to resolve tilt-translation ambiguity in goldfish

**DOI:** 10.3389/fneur.2024.1304496

**Published:** 2024-05-07

**Authors:** Shin Tadokoro, Yusuke Shinji, Toshimi Yamanaka, Yutaka Hirata

**Affiliations:** ^1^Department of Robotic Science and Technology, Graduate School of Engineering, Chubu University, Kasugai, Japan; ^2^Department of Otolaryngology, Head and Neck Surgery, National Defense Medical College, Tokorozawa, Japan; ^3^Japan Air Self-Defense Force, Ichigaya, Japan; ^4^Department of Computer Science, Graduate School of Engineering, Chubu University, Kasugai, Japan; ^5^Center for Mathematical Science and Artificial Intelligence, Chubu University, Kasugai, Japan; ^6^Academy of Emerging Sciences, Chubu University, Kasugai, Japan

**Keywords:** spatial orientation, Kalman filter model, state space model, vestibulo-ocular reflex, translation ambiguity, gravito-inertial axis, adaptive learning ability, somatogravic illusion

## Abstract

**Introduction:**

Spatial orientation refers to the perception of relative location and self-motion in space. The accurate formation of spatial orientation is essential for animals to survive and interact safely with their environment. The formation of spatial orientation involves the integration of sensory inputs from the vestibular, visual, and proprioceptive systems. Vestibular organs function as specialized head motion sensors, providing information regarding angular velocity and linear acceleration via the semicircular canals and otoliths, respectively. However, because forces arising from the linear acceleration (translation) and inclination relative to the gravitational axis (tilt) are equivalent, they are indistinguishable by accelerometers, including otoliths. This is commonly referred to as the tilt - translation ambiguity, which can occasionally lead to the misinterpretation of translation as a tilt. The major theoretical frameworks addressing this issue have proposed that the interpretation of tilt versus translation may be contingent on an animal’s previous experiences of motion. However, empirical confirmation of this hypothesis is lacking.

**Methods:**

In this study, we conducted a behavioral experiment using goldfish to investigate how an animal’s motion experience influences its interpretation of tilt vs. translation. We examined a reflexive eye movement called the vestibulo-ocular reflex (VOR), which compensatory-rotates the eyes in response to head motion and is known to reflect an animal’s three-dimensional head motion estimate.

**Results:**

We demonstrated that the VORs of naïve goldfish do not differentiate between translation and tilt at 0.5 Hz. However, following prolonged visual-translation training, which provided appropriate visual stimulation in conjunction with translational head motion, the VORs were capable of distinguishing between the two types of head motion within 3 h. These results were replicated using the Kalman filter model of spatial orientation, which incorporated the variable variance of process noise corresponding to the accumulated motion experience.

**Discussion:**

Based on these experimental and computational findings, we discuss the neural mechanism underlying the resolution of tilt-translation ambiguity within a context analogous to, yet distinct from, previous cross-axis VOR adaptations.

## 1 Introduction

The ability of animals to safely interact with the surrounding physical environment is crucial for their survival. For example, efficiently avoiding obstacles while chasing prey or escaping predators is essential for life. The accurate formation of spatial orientation is necessary to facilitate these behaviors. Spatial orientation refers to the estimation of one’s relative location, posture, and motion within a given space (1) and is created by integrating information from multiple sensors, including visual, vestibular, and proprioceptive systems. Among these, two sets of vestibular organs, namely the semicircular canals and otoliths, are specialized for sensing head motions in three-dimensional (3D) space. The semicircular canals are generally considered gyroscopes that sense angular head velocity, whereas the otoliths are accelerometers that detect gravito-inertial acceleration (GIA) (2, 3), which is the vector sum of linear head acceleration and gravitational acceleration. Consequently, it is not possible to distinguish between linear head motion (translation) and head-tilting motion relative to gravity (tilt) solely from the otolith output. This issue has been recognized as tilt-translation ambiguity, which causes somatogravic illusions that have caused several fatal aviation accidents (4, 5). Experimental studies in monkeys have confirmed that primary otolith afferents respond indistinguishably to sinusoidal translation and tilt motions when the trajectories of the two stimuli match (6, 7). The neural system must resolve this ambiguity to accurately perceive ongoing head motions and form the correct spatial orientation. Interestingly, studies on both humans and non-human primates have demonstrated that perceptions of translation and tilt are not always accurate, leading to instances of spatial disorientation. Two major theories have been proposed to explain how the human neuronal system estimates translation and tilt motion from an ambiguous otolith response (8). The frequency segregation theory posits that low-frequency linear accelerations are interpreted as tilts, whereas high-frequency accelerations are correctly interpreted as translations (9–11). In contrast, the integration theory, often referred to as the internal model theory, assumes that the brain utilizes information from multiple sensory sources, e.g., the semicircular canals, vision, and proprioception, to distinguish between these two types of motion (2, 12–14). Notably, both theories postulate the existence of an underlying neural mechanism that enables animals to distinguish different types of head motions based on their own motion experiences (2, 9, 15). However, despite these theoretical frameworks, there are limited experimental studies that explicitly address how animals adaptively learn to estimate head motion from ambiguous vestibular information based on their motion experiences.

This study explored whether animals adaptively change their head motion estimation in response to accumulated motion experiences, with a specific focus on tilt-translation ambiguity. We conducted behavioral experiments using goldfish and evaluated their vestibulo-ocular reflex (VOR) as an indicator of 3D head motion estimates. Goldfish were selected as our model species because of the comprehensive understanding of their neuroanatomy and the physiology underpinning various oculomotor behaviors (16, 17). VOR is an ultrashort latency reflexive eye movement that functions to rapidly stabilize the visual images of the retina during head motion by compensatory eye movements during which the eyes rotate in orbit based on 3D head motion estimates (see section 4.3).

We demonstrated that naïve goldfish produce a vertical VOR in response to both interaural linear acceleration and roll-tilt around their anteroposterior axis at 0.5 Hz, suggesting that their VOR does not distinguish between translation and tilt at 0.5 Hz. For goldfish with relatively more lateralized eye placement, the vertical VOR effectively stabilizes retinal images during roll-tilt head motion, whereas the horizontal VOR is more effective in stabilizing the frontal visual field during interaural translation. However, following prolonged (3 h) exposure to the same linear translational motion combined with a visual stimulus that moved parallel to the head motion, the vertical VOR response to tilt transitioned into a horizontal VOR response to tilt. This shift indicates that a correction occurred in the head motion estimate from a false tilt to an accurate translation. These results provide evidence of an adaptive process in head motion estimation that is driven by accumulated motion experience, which aligns with the predictions of the major theories regarding tilt-translation discrimination. To acquire further computational understanding of this adaptive process, we extended the Kalman filter model of spatial orientation formation originally developed by Laurens and Angelaki (18) and replicated the changes in the 3D VOR that we observed in the goldfish following visual-translation training.

## 2 Materials and methods

Procedures for animal preparation were adopted from those previously described (19–23). All relevant guidelines were followed for the use and care of animals in this study, and the Animal Welfare Committee of Chubu University approved all experimental and surgical procedures (Approval ID: 202210010).

### 2.1 Subjects

Goldfish (*Carassius auratus*) of both sexes, 10–15 cm long, were obtained from an authorized supplier (Maru-u, Japan). Thirteen goldfish were used in this study. They were maintained in a separated home aquarium at 25°C on a 12 h light/dark cycle and the water quality was monitored biweekly.

Their spindle-shaped bodies, which may have prevented naïve goldfish from accumulating linear acceleration along the interaural axis, were also advantageous for our investigation to compare their behavior before and after exposure to linear acceleration.

### 2.2 Surgical procedures

The goldfish were anesthetized by immersion in a solution 1:20.000 wt/vol of MS222 (tricaine methanesulfonate, A5040; Sigma-Aldrich, United States). A headpost set up with two screws and a pedestal of dental acrylic cemented to self-tapping screws was fastened to the frontal bones to provide head stabilization during the experiments (19).

### 2.3 Experimental setup

#### 2.3.1 Water tank

**Figure 1:**
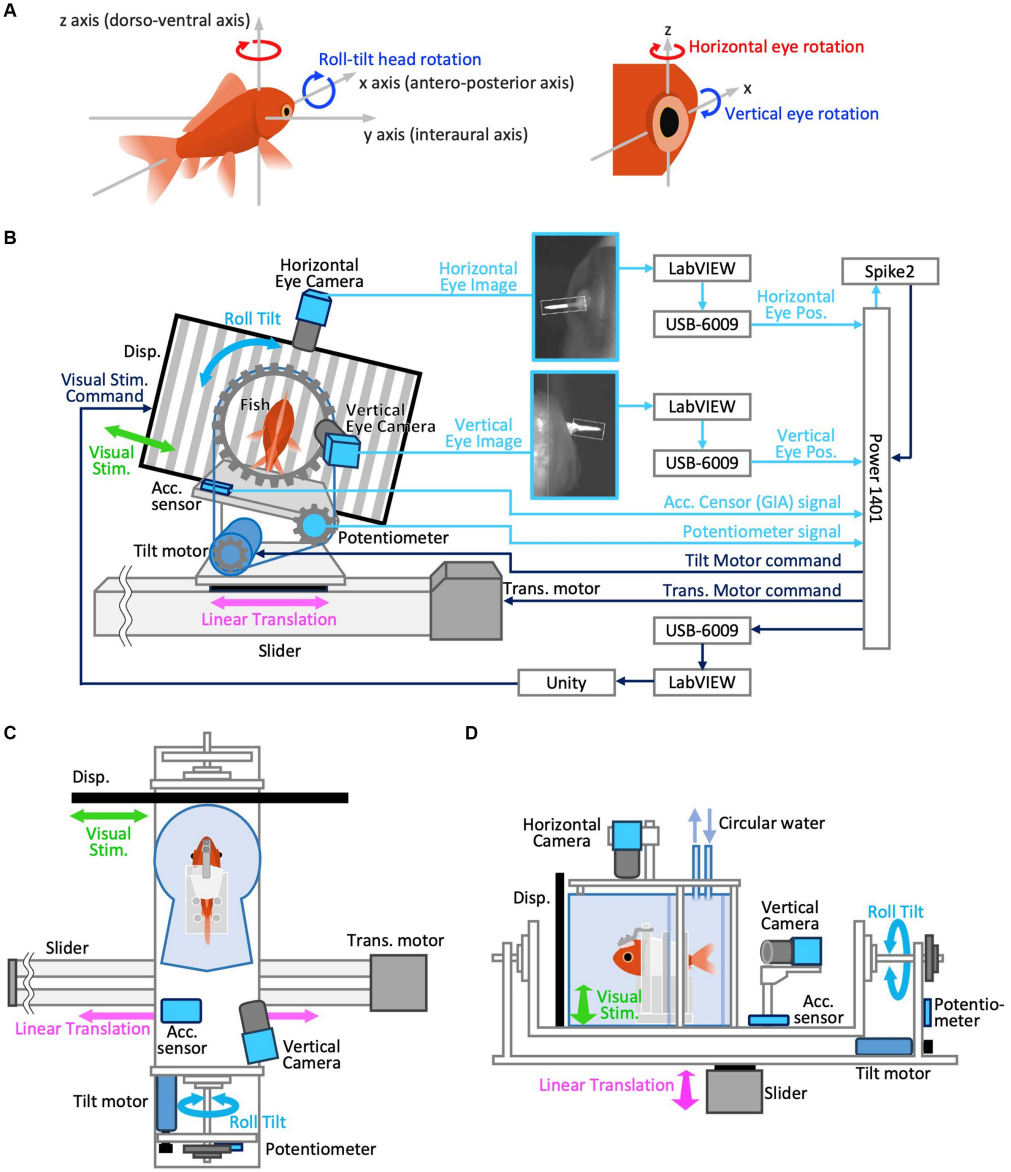
**(A)** Definition of 3D axes in the animals’ head coordination. Positive directions are indicated by arrow heads for both linear and rotational motion. To clarify, yaw and roll-tilt rotations are those around *z* and *x*-axis, respectively, which typically induce horizontal and vertical VOR, respectively. Positive directions of yaw (horizontal) and roll (vertical) rotations are clockwise, i.e., rightward in both eyes and downward in the right eye, respectively. **(B–D)** Experimental setup. **(B)** Rear view of the system (left) and its configuration diagram (right). The aquarium is removed in this view. Note, visual stimulus and linear translation were not given during actual roll-tilt test paradigm. **(C)** Top view. Horizontal eye camera is removed in this view. **(D)** Left side view.

A keyhole-shaped aquarium was designed for the experiments (Figure 1C). It comprised clear and transparent plexiglass to effectively transmit external visual stimuli. The cylindrical part of the tank was designed to ensure that, when the fish was in the center of the tank, the angle between the eyes of the goldfish and the tank wall remained consistently perpendicular. This design maintained a 0-degree angle of incidence for the light reaching the goldfish’s eyes, thereby preventing excessive refraction. Had the forward wall of the tank been planar, even if transparent, beyond the critical angle (49° for water and air), visual stimuli on the display would not reach the goldfish’s eyes. This would result in undesirable visual stimulation, as the image within the tank would reflect like a mirror. Hence, it was necessary for the tank to be cylindrical. Using an endoscope, we confirmed that the display from outside the tank does not appear distorted from the center of the cylinder. Goldfish were comfortably fixed using a headpost at the center of the cylindrical part of the aquarium. The centers of the bilateral semicircular canals were carefully positioned at the center of the cylinder. Aerated water at 25°C was filled in the sealed aquarium and circulated to appropriately maintain the animals throughout the experiment.

#### 2.3.2 Vestibular and visual stimulation

##### 2.3.2.1 Stimulus coordination

The definition of 3D axes in the animals’ head coordination, corresponding to vestibular and visual stimulus coordination (as our animals’ heads were restrained in a water tank that was securely fixed on the visual-vestibular stimulation apparatus) used in the current study, is illustrated in Figure 1A. Arrowheads indicate positive directions for both linear and rotational motions. The rotational axes of the eyes were parallel to those of the head (Figure 1A, right panel). Yaw and roll-tilt head rotations occur around the *z*- and *x*-axes, respectively, which typically induce horizontal and vertical VOR in goldfish, respectively. Positive horizontal (measured in the left eye) and vertical (measured in the right eye) movements were rightward and downward, respectively (see section 2.3.3).

##### 2.3.2.2 Vestibular stimulation

A custom-made tilt-rotation platform consisting of a rotation table connected to a DC motor (tilt motor) via a timing belt (Figures 1B–D) was used to generate the roll-tilt vestibular stimulation. The aquarium was securely attached to the rotation table, such that the anteroposterior axis (*x*-axis) of the goldfish was aligned with the rotation axis of the device. The entire tilt-rotation platform was fixed on a slider-type actuator (Slider, EZSHM6H080AZMC; Oriental Motor, Japan) driven by a stepping motor (Translation motor, AZM66MC; Oriental Motor, Japan) such that its rotation axis was orthogonal to the direction of the slider motion (Figures 1C,D). The commands to drive the tilt and translation motors were generated using Spike2 software (Cambridge Electronic Design, United Kingdom) and provided via the Power1401 interface. A potentiometer (CP-45H; Midori Precision, Japan) attached to the tilt-rotation axis was used to measure the tilt angle of the rotation table. An acceleration sensor (KXR94-2050; Akizuki Denshi Tsusho, Japan) installed on the rotation table measured the GIA component along the interaural axis (*y*-axis) of the fish.

##### 2.3.2.3 Visual stimulation

The optokinetic stimulus (OKS) was presented on a 13.3-in liquid-crystal display monitor with a refresh rate of 60 Hz (KNH133-133-ZH; Shenzhen Kenuohua Electronics, China) fixed on a slider and positioned 70 mm in front the center of the cylindrical section of the aquarium (Figure 1C). The monitor covered approximately 129° of the animal’s visual field. The center of the cylinder corresponded to the location of the animal’s semicircular canals and was close to the eyes. The OKS was created using the Unity game engine (Unity Technologies, United States). The OKS velocity command was generated using LabVIEW software (National Instruments, United States) and transferred to Unity via Transmission Control Protocol/Internet Protocol communication to move the vertical black-and-white stripes. OKS moved sinusoidally in synchrony with the translational vestibular stimulation at the same frequency (0.5 Hz), with a maximum amplitude of 38°/s. For example, the OKS moves rightward during the leftward movement of the slider. Presented on a flat display aligned parallel to the *y*-axis, the OKS physically traversed along the *y*-axis, manifesting its motion as a linear velocity; hence, no visual motion was observed along the *x*-axis. The angular velocity at 38°/s, which is the maximum amplitude of the OKS velocity, is equivalent to 0.055 m/s as a linear velocity along the *y*-axis at the direct front of the goldfish on the display. This visual scene was intended to convey to the goldfish the direction of its actual physical self-motion during translational motion. While considerably slower in comparison to the maximum linear velocity of translational motion (0.64 m/s, as detailed in section 2.4), this deliberate speed adjustment was implemented due to concerns that an excessively rapid OKS might be imperceptible to the goldfish. Additionally, the choice was influenced by the limitation of the liquid-crystal display monitor with a refresh rate of 60 Hz, which may not smoothly render the OKS. Due to the possibility of independent software (Spike2 and LABVIEW) potentially generating stimulation signals slightly out of phase, we synchronized OKS motion with vestibular stimulation by having the LabVIEW software receive a copy of commands for the vestibular apparatus (tilt motor or translation motor) from Spike2 via a USB-6009 data acquisition (DAQ) device (National Instruments). For the experiments conducted in the dark (section 2.4), the OKS display was turned off to obtain complete darkness.

#### 2.3.3 Eye movement recording

The rotational axes of the eye used in this study are parallel to those of the head. Horizontal and vertical eye movements are rotations around the *z*- and *x*-axes, respectively, passing through the eye’s center of rotation (Figure 1A). Vertical VOR eye movements are induced by head rotation around the *x*-axis in lateral-eyed animals such as goldfish, whereas the same vestibular stimulation induces torsional VOR in front-eyed animals such as primates. Horizontal and vertical eye movements were extracted from video images acquired at 601 fps using two CMOS cameras (DMK33UX273; Imaging Source, Germany) equipped with infrared filters (IR-86; Fujifilm Corporation, Japan). The eyes were illuminated using an infrared light source (AE-LED56; Akizuki Denshi Tsusho, Japan) to capture clear images (Figure 1B). The camera to measure horizontal eye movements was placed atop the aquarium to capture the left eye, whereas the camera to measure vertical eye movements was placed behind the aquarium at a slightly oblique position (10° to the right) to capture the right eye. This inclination ensured an unobstructed view of the goldfish eye movements from the rear (Figure 1C). It has been demonstrated and reconfirmed that goldfish binocular eye movements are conjugated during VOR and that their eye velocity traces are identical (19). Positive horizontal (left eye) and vertical (right eye) eye movements occurred in the rightward and downward directions, respectively (Figure 1A).

To accurately measure the eye rotation angles, a small cone-shaped marker, coated with thermoplastic containing titanium dioxide, was attached to each eye. It was affixed to a peripheral part of the cornea outside the edge of the pupil (Figure 1B) using a cyanoacrylate adhesive after the administration of surface anesthesia using lidocaine, just before the goldfish was placed in the aquarium.

The images acquired from the two cameras were individually sent to two different personal computers (PCs) via USB 3.0, and the eye positions (rotation angles) were measured at 200 Hz using custom-made software developed using the LabVIEW Vision Toolkit (National Instruments). The zero eye position (0°) was set to the null eye position, where the eyes asymptotically drift back after spontaneous saccades in the dark (24–27). Corrections for the vertical eye position obtained from a 10° oblique camera were applied to the data (see Supplementary Methods, section 2.3.3.1). A potentially slight tilt of the horizontal eye camera away from the *z*-axis can detect an erroneous horizontal component arising from purely vertical eye movements. Corrections for the horizontal eye position data are also provided (see Supplementary Methods, sections 2.3.3.2 and 2.3.3.3).

Horizontal and vertical eye position data extracted from the separate PCs were converted to voltage signals via the respective DAQ device (USB-6009 interface) connected to the respective PCs and transferred to Power1401 to record them in synchrony with other sensor data at a sampling frequency of 1,000 Hz in 16-bits in the Spike2 program (Figure 1B).

### 2.4 Experimental paradigms

**Figure 2:**
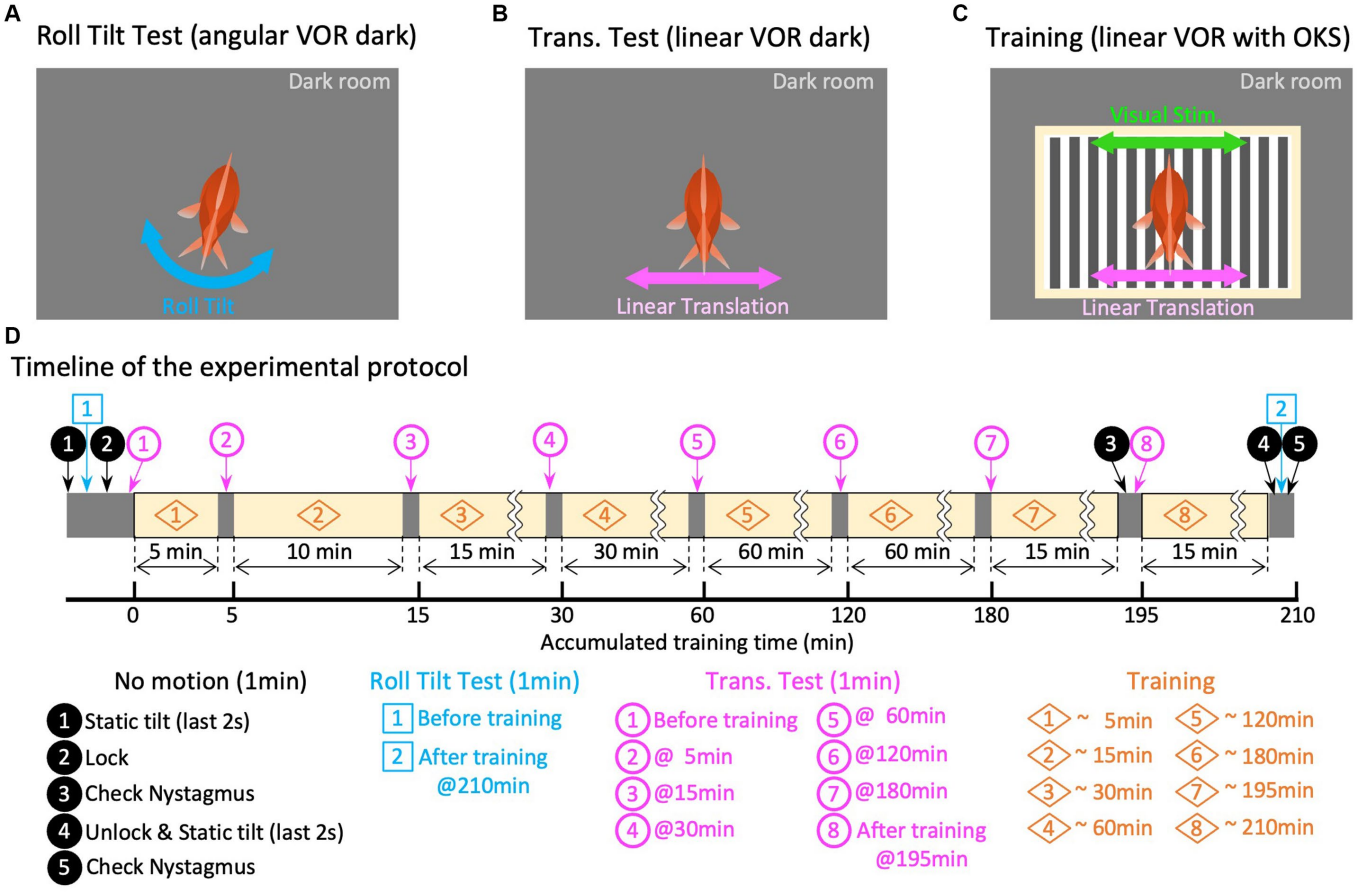
Experiment paradigm. Panels **(A–C)** are graphical explanations of the roll-tilt test (angular VOR in the dark), translation test (linear VOR in the dark), and training (linear VOR along with OKS), respectively. **(D)** Schematic overview of the timeline of the experimental protocol. The times in the titles of each paradigm of translation test and training are the accumulated times of training experienced up to that point, which differs from the overall elapsed time because of the test segments inserted before and during training.

All animals were subjected to identical experimental paradigms, as summarized in Figure 2D. After preparing for recording, the animals were acclimated to the apparatus for 60 min before the experimental intervention. The total measurement time per animal was 225 min (210 min for training, 8 min for the translation test, 2 min for the roll-tilt test, and 5 min for no motion). The swimming behavior of all animals was carefully inspected for at least 30 min after the experiment to confirm their normal behavior.

Before training, 1 min of eye movement observation (no motion) was conducted in the dark without any stimulation to confirm the absence of abnormal eye movements, such as nystagmus. During the last 2 s of “no motion,” the initial tilt position was gradually shifted to 12.7° in a clockwise manner to provide symmetrical tilt-rotation stimulation about the earth’s vertical axis (*z*-axis). From this initial position, a roll-tilt rotational stimulus was applied for 1 min in the dark to evaluate the roll-tilt VOR in the animal’s naïve state (Figures 2A,D, Roll-Tilt Test). The angular velocity of the roll-tilt stimulation was a sinusoid at 0.5 Hz, with maximum speed and acceleration of 40 °/s and 0.22 G, respectively. Subsequently, another “no motion” period was allowed for 1 min, during which the tilt-rotation table was locked to the slider to prevent unintended rotation during the subsequent translation stimulations. A linear translational stimulus was then applied in the dark for 1 min to evaluate the translational VOR in the naïve state (Figures 2B,D, Translation Test, magenta circled number 1). The velocity of the translation stimulation was sinusoid at 0.5 Hz; with a maximum linear speed and acceleration of 0.64 m/s and 0.2 G, respectively.

Then, the training and test paradigms were conducted for a total of 218 min (Figures 2C,D, Training). During the training period, the same linear translational stimulus as that in the Translation Test was provided with the OKS, which moved synchronously with the translation velocity (see section 2.3.2.3). Seven translation tests, each lasting 1 min, were performed during the training and test paradigms at 5, 15, 30, 60, 120, 180, and 195 min (Figure 2D, magenta circled numbers 2–8). The no motion period was imposed before the final translation test (Figure 2D, magenta number 8) to observe the eye movements immediately after training. After completing all the training periods, the rotation table was unlocked during another 1-min “no motion” observation, followed by roll-tilt testing to evaluate the roll-tilt VOR after training (Figures 2A,D, Roll-Tilt Test). Finally, “no motion” observations were conducted in the dark to confirm the absence of abnormal eye movements.

Because this study focuses on exploring the potential selective adaptability of the otolith-ocular system and the motion estimation system, particularly focusing on their ability to achieve visually guided reinterpretation of translation and tilt, we selected 0.5 Hz as the frequency of sinusoidal stimuli. In primates, translational head motion along the *y*-axis induces a gradual shift in the VOR axis from torsional to horizontal in the intermediate-frequency range of approximately 0.1–1 Hz (28, 29). During translational head motion along the *y*-axis at low frequencies (< 1/7 Hz) in goldfish, a vertical VOR was observed around the *x*-axis (30). Therefore, we concluded that a frequency of 0.5 Hz was appropriate for our sinusoidal stimuli, as it is within the intermediate frequency range where the shift in the VOR axis can be observed. Notably, this frequency aligns with the frequency used in a previous translational cross-axis VOR adaptation study (31), which aimed to evaluate the adaptive changes in motion interpretation during translation (see section 4.1).

### 2.5 Data analysis

All the data recorded at a sampling rate of 1,000 Hz were anti-aliased using an 11-point moving average filter with a cut-off frequency of 40.27 Hz and then resampled at 100 Hz for offline analysis. After downsampling, the high-frequency noise components in the vestibular stimuli were eliminated by applying a 21-point moving average filter twice with a cut-off frequency of 2.11 Hz. Similarly, the high-frequency noise components in the eye movement recordings were eliminated by applying an 11-point moving average filter twice, with a cut-off frequency of 4.03 Hz.

#### 2.5.1 Calculation of linear velocity, tilt angular velocity, and equivalent GIA angular velocity

From the recorded vestibular stimulus data (linear acceleration along the *y*-axis and roll-tilt rotational angle around the *x*-axis), linear velocity, tilt angular velocity, and the velocity of GIA vector rotation (GIA angular velocity) were calculated as follows.

**Figure 3:**
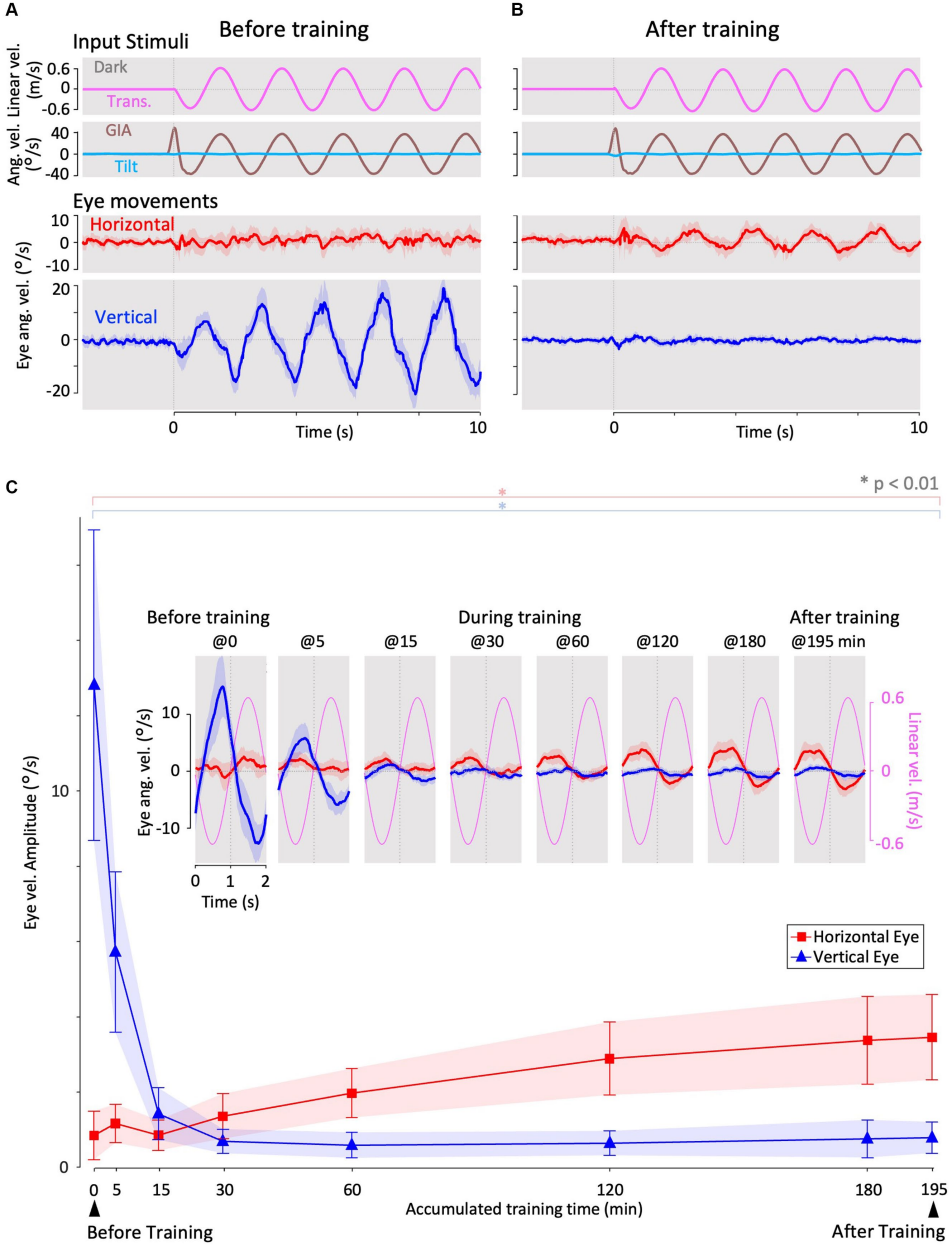
Horizontal and vertical VOR in response to interaural translational head acceleration in the dark (translation test) before, during, and after visual-translation training. **(A,B)** Horizontal (second panels from the bottom, red) and vertical (bottom panels, blue) eye velocities, around the *z*- and *x*-axes respectively, averaged over all animals (*n* = 12) during translational acceleration stimulation in the dark before **(A)** and after **(B)** training. Red and blue shadows indicate plus or minus one standard deviation. The top panels show linear velocity along the *y*-axis converted from the acceleration stimulation (see section 2). The second panels from the top show tilt (rotation around the *y*-axis) angular velocity, which is 0 during this stimulation and GIA angular velocity calculated from the translational acceleration, both around the *x*-axis. **(C)** Inset illustrating the horizontal (red) and vertical (blue) eye velocity traces averaged over stimulus cycles (magenta). The angular velocity traces around different axes (horizontal eye velocity around the *z*-axis and vertical eye velocity around the *x*-axis) and linear velocity along the *y*-axis traces are depicted in the same figures. Below the insets, changes in the mean amplitudes of horizontal and vertical VORs (learning curves) are plotted. Red and blue shadows indicate plus or minus one standard deviation. The increase in mean (±SD) amplitude of horizontal eye velocity and the decrease in mean amplitude of vertical eye velocity before and after training are significant (0.3 ± 0.6 vs. 3.4 ± 1.1 °/s, *p* < 0.001 for horizontal eye velocity; 12.5 ± 4.1 vs. 0.7 ± 0.4 °/s, *p* < 0.001 for vertical eye velocity).

**Figure 4:**
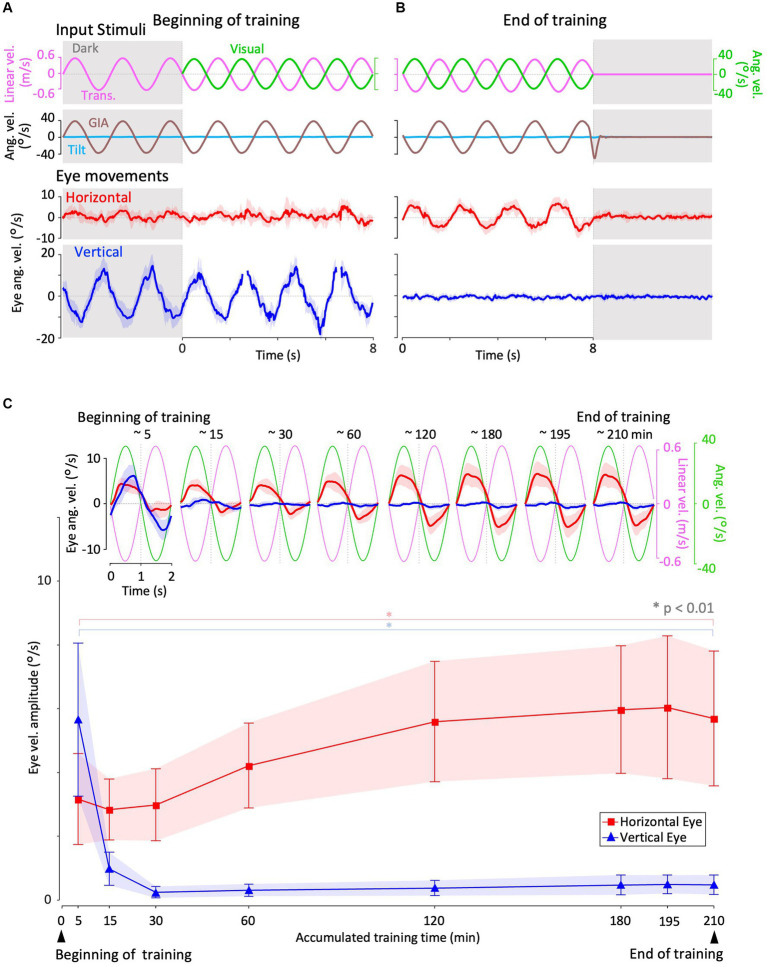
Horizontal and vertical VORs in response to interaural translational head acceleration along with OKS (training) during visual-translation training. **(A,B)** Horizontal (second panels from the bottom, red) and vertical (bottom panels, blue) eye velocities around the *z*- and *x*-axes, respectively, averaged over all animals (*n* = 12) during translational acceleration stimulation along with OKS at the beginning **(A)** and end **(B)** of the training. Red and blue shadows indicate plus or minus one standard deviation. The top panels show linear velocity along the *y*-axis converted from the acceleration stimulation (see section 2), and OKS velocity represented as an angular velocity around the *z*-axis. The second panels from the top show tilt angular velocity, which is 0 during this stimulation and GIA angular velocity calculated from the translational acceleration, both around the *x*-axis. **(C)** Inset illustrating horizontal (red) and vertical (blue) eye velocity traces averaged over stimulus cycles (magenta). The angular velocity traces around different axes (horizontal eye velocity and visual stimulus velocity both around the *z*-axis, vertical eye velocity around the *x*-axis) and linear velocity along the *y*-axis traces are depicted in the same figures. Eye velocities and visual stimulus velocity are depicted in different scales. Below the insets, changes in the mean amplitudes of horizontal and vertical VORs (learning curves) are plotted. Red and blue shadow indicate plus or minus one standard deviation. The increase in mean (±SD) amplitude of horizontal eye velocity and the decrease in mean amplitude of vertical eye velocity between the beginning and end of the training are significant (3.1 ± 1.4 vs. 5.6 ± 2.1 °/s, *p* = 0.004 for horizontal eye velocity; 5.6 ± 2.3 vs. 0.4 ± 0.3 °/s, *p* < 0.001 for vertical eye velocity).

**Figure 5:**
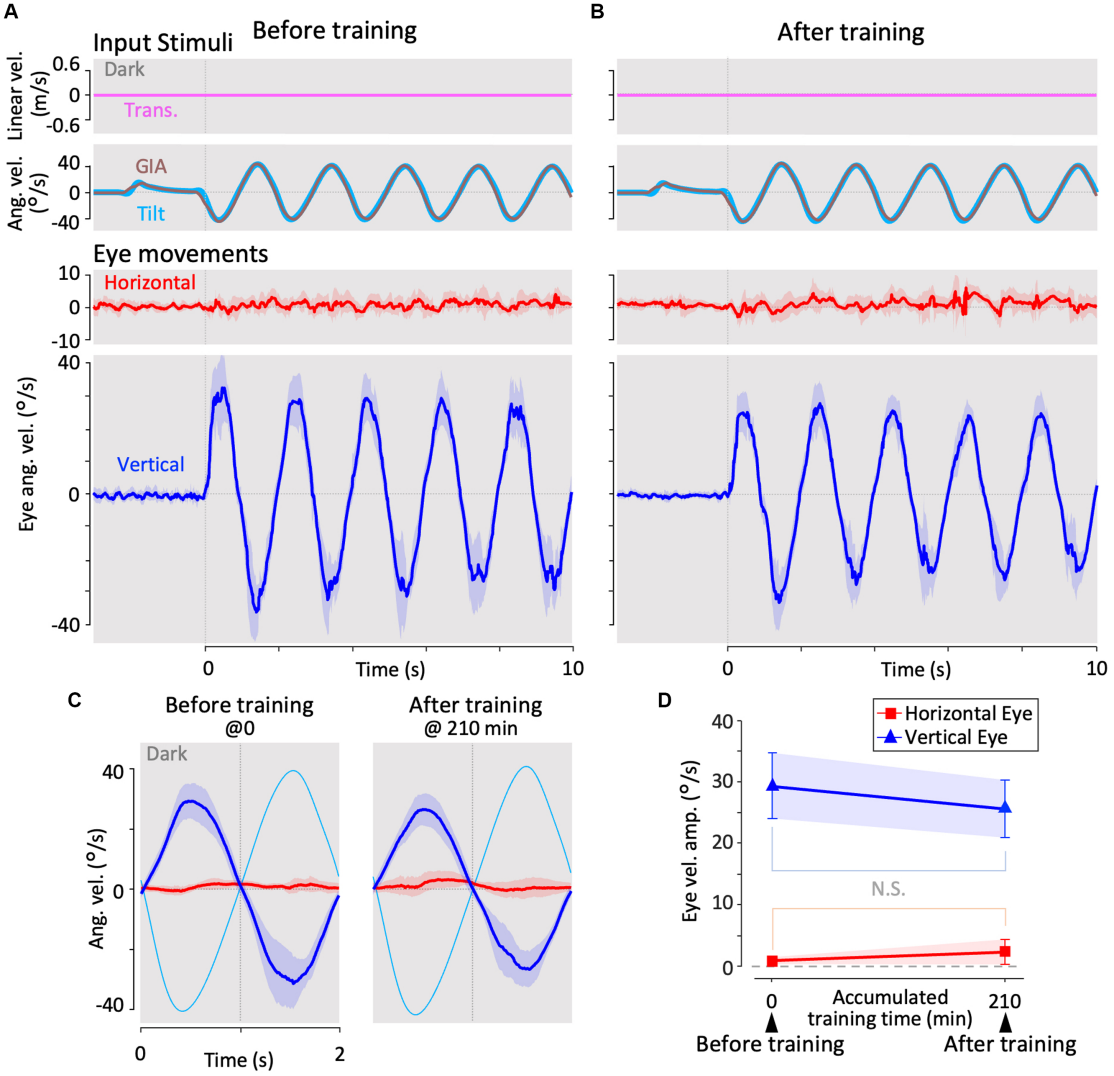
Horizontal and vertical VOR in response to roll-tilt head rotation in the dark (roll-tilt test) before, and after visual-translation training. **(A,B)** Horizontal (second panels from the bottom, red) and vertical (the bottom panels, blue) eye velocities around the *z*- and *x*-axes respectively, averaged over all animals (*n* = 12) during roll-tilt stimulation in the dark before **(A)** and after **(B)** training. Red and blue shadows indicate plus or minus one standard deviation. The top panels show linear velocity along the *y*-axis, which is 0 during this stimulation. The second panels from the top show tilt angular velocity and GIA angular velocity both around the *x*-axis. **(C)** Horizontal (red) and vertical (blue) eye velocity traces averaged over stimulus cycles (cyan). Angular velocity traces around different axes (horizontal eye velocity around the *z*-axis, vertical eye velocity and tilt angular velocity both around the *x*-axis) are depicted in the same figures. **(D)** Changes in the mean amplitudes of horizontal and vertical VORs (learning curves). Red and blue shadows indicate plus or minus one standard deviation. Both horizontal and vertical eye velocities show no significant differences between before and after training (0.6 ± 0.6 vs. 1.3 ± 2.0 °/s, *p* = 0.157 for horizontal eye velocity; 29.1 ± 5.4 vs. 25.4 ± 4.7 °/s, *p* = 0.060 for vertical eye velocity).

Linear velocity during linear translation was calculated by integrating the *y*-axis data of the acceleration sensor using the MATLAB (MathWorks, United States) “cumtrapz” function (Figures 3, 4). The tilt angular velocity was calculated by differentiating the potentiometer angle data using a three-point low-pass differentiation filter (Figure 5). To obtain GIA angular velocity, we first calculated the rotational angle of the GIA vector relative to the earth’s vertical axis (*z*-axis), which rotates around the goldfish’s antero-posterior axis (*x*-axis) using the arctangent of acceleration as $\theta_{GIA}=\tan^{-1}\left(Acc.\mathrm{Sensor}\;\mathrm{Value}\right)$ during translational motion following Lichtenberg et al. (32) and arcsine of acceleration as $\theta_{GIA}=\sin^{-1}\left(Acc.\mathrm{Sensor}\;\mathrm{Value}\right)$ during the roll-tilt motion (see Supplementary Methods, section 2.5.1). The GIA angular velocity was obtained by differentiating the rotational angle of the GIA vector using a three-point low-pass differentiation (Figures 3–5). High-frequency noise components amplified by differentiation processing in the vestibular stimuli were eliminated again by applying a 21-point moving average filter twice, with a cut-off frequency of 2.11 Hz. For the GIA angular velocity, the counterclockwise direction was defined as positive to better illustrate its relationship with the tilt angular velocity. The GIA angular velocity was presented to show that translational acceleration stimulation (Figures 3, 4) and roll-tilt stimulation (Figure 5), which are currently used, generated almost identical stimulation to that of otoliths.

#### 2.5.2 Eye velocity evaluation

Horizontal and vertical eye velocities were obtained by differentiating the eye position data using a three-point low-pass differentiation. High-frequency noise components amplified by the differentiation processing of eye velocities were eliminated by applying an 11-point moving average filter twice with a cutoff frequency of 4.03 Hz. To evaluate the VOR, saccades and high-frequency noise, if present, were eliminated from the horizontal and vertical eye position data by applying a custom-made automatic desaccading algorithm. The eliminated portions of the data were excluded from further analyses. The eye velocity traces from all individual animals were time-aligned and averaged throughout the experiment, as they all underwent identical experimental sequences. Notably, because of desaccade processing, the averaging processing at each time point was not always by the same number of samples, especially immediately after sudden vestibular or visual stimulation following the no-motion period, where eye velocity tended to be unstable due to startle-like responses.

For quantitative evaluation, individual eye velocity data were averaged over stimulus cycles for the entire 1-min test period and for the last 5 min of the training periods. Sinusoidal curve fits were performed to obtain estimates of amplitude and phase from these individually averaged VOR eye velocity data by using the MATLAB “fittype” function where frequency was fixed to 0.5 Hz (stimulus frequency). The individually averaged eye velocity data and estimated amplitudes were averaged across all individuals. The amplitudes of the horizontal eye velocity during the roll-tilt test and first translation test paradigms were not well approximated because of their minimal and unstable horizontal eye movement. This lack of stability may have been influenced by both the small magnitude of these eye velocities and startle-like responses following sudden vestibular stimulation after the “no motion” period.

Differences in the estimated eye velocity amplitudes between the first and last paradigms of the translation test, roll-tilt test, and training were evaluated using the Wilcoxon rank-sum test, along with individually estimated eye velocity amplitudes.

### 2.6 Kalman filter model

Laurens and Angelaki (18) constructed a three-dimensional Kalman filter model. The Kalman filter represents the simplest and most commonly used mathematical technique to implement statistically optimal dynamic estimation and explicitly computes sensory prediction errors. We employed a modified version of their model for spatial orientation formation to interpret our results. This model was configured such that active and passive head movements were treated as inputs to the system and process noise was added to the input. We revised the model to simulate VORs in response to translation and tilt head motion in goldfish (lateral-eyed animals) based on estimates of 3D head motion and visual stimulation (OKS) states. Moreover, we introduced the estimated state of gravity ( $\hat{G}$ ) from the previous time step, obtained using the Kalman filter, into the Kalman filter gain matrix. This enabled us to describe the calculation of state estimates realistically, addressing the issue posed by Laurens’ model regarding the calculation of state estimates using the real state of gravity acceleration ( G ), which the brain cannot inherently perceive. Notably, this modification simultaneously rendered our model nonlinear. The validity of this nonlinearity will be discussed in section 4.2. In the revised model, the states to be estimated by the Kalman filter algorithm are head linear acceleration A , head linear velocity B , head rotation velocity Ω , gravitational acceleration G , eye rotation velocity P , visual rotation velocity Φ , and semicircular canals endolymph rotation velocity C (all 3D vectors are summarized in Tables 1, 2). For the sensors, the model incorporated semicircular canals, otoliths, and the retina, similar to the Laurens and Angelaki model (18). We assumed that, when animals looked forward (see section 4.3), the reflexive eye velocity around each axis was generated in the opposite direction to the head rotation velocity and head linear velocity states and in the same direction as the visual rotation state, according to the following equations.


(1) $$P_{x}^{u}\left(t\right)=h\left[{\hat{\Phi }}_{x}\left(t\right)-{\hat{\Omega }}_{x}\left(t\right)\right]$$

(2) $$P_{y}^{u}\left(t\right)=h\;\left[{\hat{\Phi }}_{y}\left(t\right)-{\hat{\Omega }}_{y}\left(t\right)+r{\hat{B}}_{z}\left(t\right)\right]$$

(3) $$P_{z}^{u}\left(t\right)=h\left[{\hat{\Phi }}_{z}\left(t\right)-{\hat{\Omega }}_{z}\left(t\right)–r{\hat{B}}_{y}\left(t\right)\right]$$

**Table 1:** Variables in the Kalman filter model.

Motion variables	
A	Head linear acceleration
Ω	Head rotation velocity
G	Gravity acceleration
B	Head linear velocity
P	Eye rotation velocity
C	Semicircular canals endolymph rotation velocity
Φ	Visual rotation velocity
Sensory variables	
F	Otolith signal
V	Semicircular canal signal
Ψ	Retina signal
Input variables and noises	
$A^{u}$	Active motion of head linear acceleration
$\Omega^{u}$	Active motion of head rotation velocity
$P^{u}$	Eye motor command
$A^{\varepsilon }$	Process noises + passive stimuli of head linear acceleration
$\Omega^{\varepsilon }$	Process noises + passive stimuli of head rotation velocity
$\Phi^{\varepsilon }$	Process noises + passive stimuli of visual rotation velocity
$P^{\varepsilon }$	Process noise of eye motor command
$F^{\eta }$	Observation noise of otolith signal
$V^{\eta }$	Observation noise of semicircular canal signal
$\Psi^{\eta }$	Observation noise of retina signal
Feedback signals and gain	
$\Delta \hat{A}$	Feedback signal to correct estimation of head linear acceleration
$\Delta \hat{G}$	Feedback signal to correct estimation of gravity acceleration
$\Delta \hat{\Omega }$	Feedback signal to correct estimation of head rotation velocity
$\Delta \hat{B}$	Feedback signal to correct estimation of head linear acceleration
$\Delta \hat{P}$	Feedback signal to correct estimation of eye velocity
$\Delta \hat{C}$	Feedback signal to correct estimation of semicircular canal dynamics
*K*	Kalman gain

**Table 2:** Simulation parameters in the Kalman filter model.

Variables		Values
Δt	Simulation time step	0.002 s
$\tau_{C}$	Canal dynamics time const	0.5 s
$\tau_{m}$	Muscle dynamics time const	0.003 s
r	Reciprocal of viewing point length	4
h	Eye control gain	0.8
$\sigma^{\Omega }$	Head rotation velocity SD of process noises + passive stimuli	40 °/s
$\sigma_{x}^{A}$	Head linear acceleration SD of process noises + passive stimuli along *x*-axis	0.001 G
$\sigma_{y}^{A}$	Head linear acceleration SD of process noises + passive stimuli along *y*-axis	0.001 G or 0.180 G
$\sigma_{z}^{A}$	Head linear acceleration SD of process noises + passive stimuli along *z*-axis	0.001 G
$\sigma^{\Phi }$	Visual rotation velocity SD of process noises + passive stimuli	1 °/s
$\sigma^{P}$	Eye velocity process noise SD	3 °/s
$\sigma^{V}$	Canal observation noise SD	3 °/s
$\sigma^{F}$	Otolith observation noise SD	0.02 G
$\sigma^{\Psi }$	Retinal slip observation noise SD	30 °/s

where $P_{x}^{u}\left(t\right)$ , $P_{y}^{u}\left(t\right)$ , and $P_{z}^{u}\left(t\right)$ represent the eye motor command $P^{u}$ around the *x*-, *y*-, and *z*-axes, respectively, as defined in Figure 1A. The coefficient h was set to 0.8 based on the rotational VOR gain of goldfish as reported in Pastor (19, 33). The coefficient r was determined to be 4 to replicate the translational VOR experiment presented in this paper. The hat operator is estimated using the Kalman filter. The sign of the vertical eye motor command $P_{x}^{u}\left(t\right)$ (i.e., up and downward) switches for each eye in lateral-eyed animals. Equation (1) applies to the right eye, as observed in this study. The structure of the model is depicted in Figure 6, and a detailed description of the model and the mathematical equations used are provided in the Supplementary Methods, section 2.6. Individuals interested in obtaining the code can contact the corresponding author via email for individual access.

**Figure 6:**
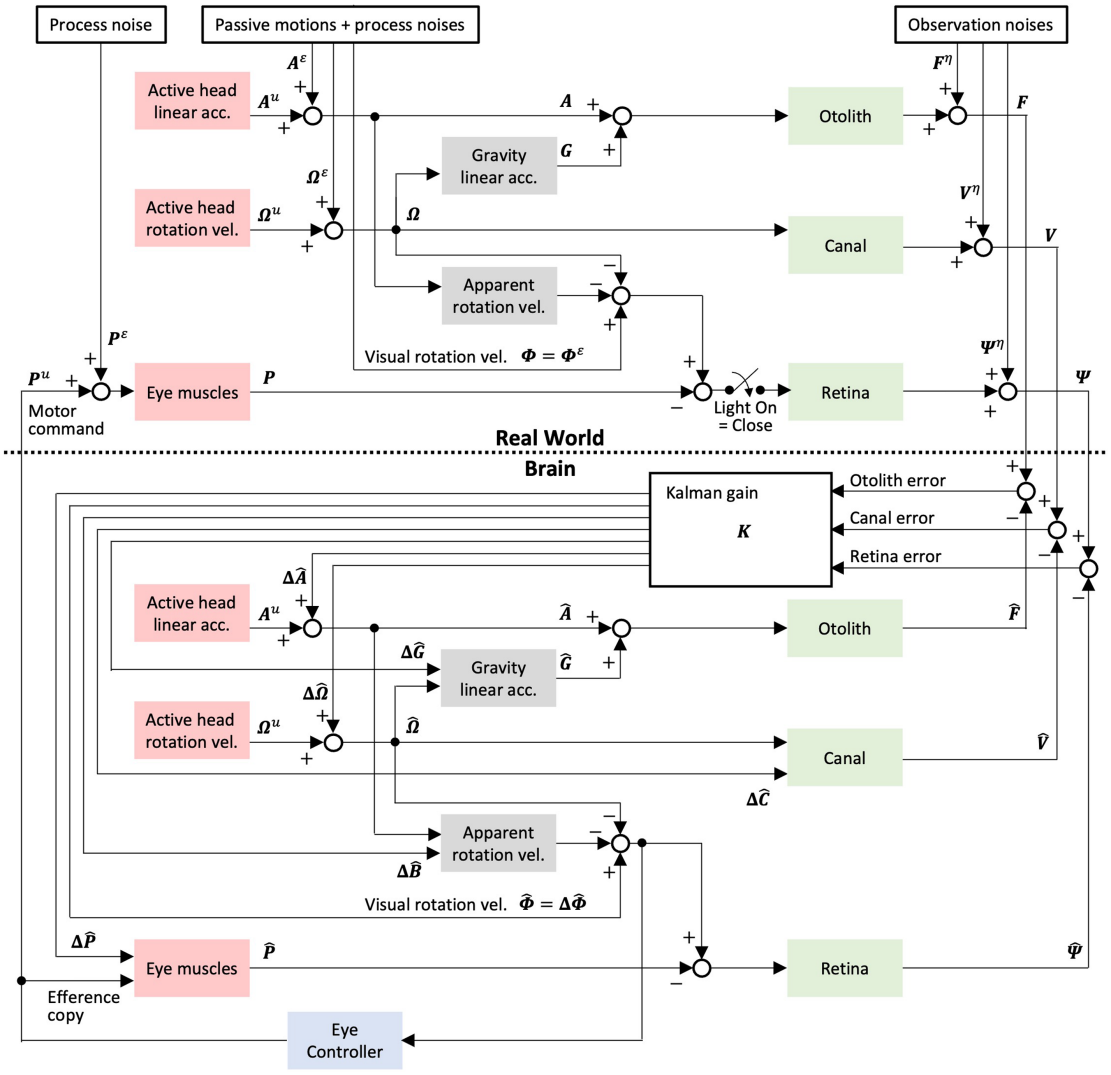
Modified Kalman filter model, where each variable and coefficient are defined in Table 1. The eye motor model, retina model, apparent rotation velocity model, and eye controller were added to the Kalman filter model in Laurens and Angelaki (18) to explain our results of VOR. The apparent rotational velocity model outputs the visual rotational velocity around the head caused by linear acceleration, which is used to linear VOR in the eye controller [Equation (1–3)]. To assist in the interpretation of our experiments, particularly with the interpretation of eye movements, passive motion, and process noise, we have also described a world system outside the brain that was not specified in the Kalman filter model by Laurens and Angelaki.

The following simulations were conducted to confirm the validity of the model and evaluate the 3D VOR induced as well as 3D head motion states estimated by the Kalman filter: angular VOR in the dark induced by the angular velocity stimuli of sinusoidal waves (frequency: 0.5 Hz, amplitude: 38°/s) around each axis, optokinetic response (OKR) induced by visual stimuli of sinusoidal wave (frequency: 0.5 Hz, amplitude: 38°/s), and optokinetic response after nystagmus (OKAN) around the *z*-axis after the visual stimuli is turned off. Subsequently, using the same set of model parameters as those used for model validation, we simulated the following: (1) translational VOR induced by the translational acceleration stimuli of sinusoidal waves (frequency: 0.5 Hz, amplitude: 0.2 G) along the *y*-axis, (2) visual-translational VOR induced by visual stimuli of sinusoidal wave (frequency: 0.5 Hz, amplitude: 38°/s) around the *z*-axis and translational acceleration stimuli of the sinusoidal wave (frequency: 0.5 Hz, amplitude: 0.2 G) along the *y*-axis, and (3) angular VOR induced by the angular velocity stimuli of sinusoidal waves (frequency: 0.5 Hz, amplitude: 38°/s) around the *x*-axis. Furthermore, to express the animal’s increased experience in translation acceleration, we increased the standard deviation (SD) of linear head acceleration input along the *y*-axis (Table 2, $\sigma_{y}^{A}$ ) from 0.001 G before training to 0.180 G after training, whereas other parameters were unchanged.

## 3 Results

Among the 13 animals used in this study, one showed unstable eye movements and VOR adaptation and was therefore excluded from further data analyses and evaluation. First, we show the interaural (*y*-axis) translational VOR in the dark for the remaining 12 animals before training (see section 3.1). We then show how translational VOR is altered by visual stimulation combined with head translation during training (section 3.2), followed by post-training translational VOR in the dark (section 3.3). We also demonstrate the tilt (around the anteroposterior *x*-axis) of the VOR tested before and after training (section 3.4). Finally, we present the simulation results of the Kalman filter model (section 3.5).

### 3.1 VOR during translation in dark in naïve animals

Figure 3A shows the horizontal (red) and vertical (blue) eye velocities averaged for all animals (*n* = 12) during translational acceleration stimulation in the dark before training (Figure 2D; magenta circle 1). Red and blue shadows indicate plus or minus one standard deviation. The top panel shows the linear velocity (along the *y*-axis) converted from acceleration stimulation (see section 2.5.1). The second panel from the top shows the tilt angular velocity, which is zero during this stimulation, and the GIA angular velocity calculated from the translational acceleration (see section 2.5.1), both around the *x*-axis. The GIA angular velocity revealed that stimulation during this paradigm and roll-tilt stimulation (shown in Figure 5) generated almost identical stimulation to that of the otoliths. Clearly, robust vertical VOR with an average amplitude of 12.5°/s was induced, whereas very small, if any, horizontal VOR was observed in naïve animals. In contrast to naïve goldfish, naïve primates have shown horizontal VOR around the z-axis during interaural translation (at 0.5 Hz) rather than around the *x*-axis (torsional VOR in the case of front-eyed animals) (9, 13, 31). The human VOR around the *x*-axis, which is similar to the results observed in our goldfish, was found at lower frequencies. Specifically, at frequencies of 1/200–1/40 and 1/5–2/5 Hz, torsional VOR (equivalent to the vertical VOR in goldfish) around the *x*-axis, rather than horizontal VOR, has been demonstrated during interaural translation in upright humans (10, 32).

These results are confirmed in a cycle average format in the inset of Figure 3C (before training at 0 min), illustrating the horizontal (red) and vertical (blue) eye velocity traces averaged over the stimulus cycles (magenta). A slight phase lag (35.7°) in reference to the stimulus velocity was noted for the vertical VOR eye velocity. Similar to the present findings in goldfish, previous results on human torsional VOR eye velocity during interaural translation also demonstrated a phase delay from the sinusoidal GIA angular velocity. For instance, in humans, this phase lag reaches approximately 22.5° at 1/200 Hz and 48.6° at 1/5 Hz (10, 32).

Before examining how these translational VORs in the dark change throughout the training process, we demonstrate the progression of the horizontal and vertical eye velocities during the training paradigm in the next section.

### 3.2 Changes in VOR during visual-translation training

Figure 4 illustrates changes in the horizontal and vertical VOR eye velocity during training, in which the same translational acceleration stimulus as in the test was combined with visual stimulation (see section 2). Figures 4A,B illustrate the horizontal (red) and vertical (blue) eye velocities for four stimulus cycles (8 s) averaged for all animals at the beginning (A) and end (B) of training. At the beginning of training, a clear vertical VOR was induced, whereas little horizontal VOR was observed. After 3 h of training, the relationship was reversed, where a robust horizontal VOR and almost no vertical VOR were induced.

The insets in Figure 4C illustrate the horizontal (red) and vertical (blue) eye velocity averaged over stimulus cycles (linear velocity in magenta, visual stimulus velocity in green) during the initial 5, 10–15, 25–30 min, and thereafter. At the beginning of the training (0–5 min), the amplitudes of the horizontal and vertical VOR eye velocity are 3.1 and 5.6 °/s, respectively. The mean amplitude of horizontal VOR increased gradually along with the training time while that of the vertical VOR rapidly decreased to almost zero in the initial 30 min of training. The increase in mean (±SD) amplitude of the horizontal eye velocity and the decrease in mean amplitude of the vertical eye velocity between the beginning and end of the training are significant (3.1 ± 1.4 vs. 5.6 ± 2.1 °/s, *p* = 0.004 for horizontal eye velocity; 5.6 ± 2.3 vs. 0.4 ± 0.3 °/s, *p* < 0.001 for vertical eye velocity).

The learning curves of the changes in horizontal and vertical VOR amplitudes during training are illustrated in the insets below. Red and blue shadows indicate plus or minus one standard deviation for the horizontal and vertical VOR amplitudes, respectively. The learning curves indicated that the initial noncompensatory VOR (large vertical and smaller horizontal eye velocities) became more compensatory (almost no vertical or larger horizontal eye velocities) to reduce retinal image slip during the training stimulus as training progressed. In the next section, we illustrate how these changes in the VOR during training are reflected in eye movements during the test stimulus in the dark.

### 3.3 Changes in VOR during translation in the dark along with the training

Figure 3B illustrates the horizontal (red) and vertical (blue) VOR eye velocities during translation in the dark after 3 h of training in the same format as in Figure 3A, showing those at the beginning of training. Notably, the horizontal VOR was robust, whereas the vertical VOR was significantly suppressed after training. The insets in Figure 3C show the averaged horizontal and vertical VOR eye velocity traces during translation stimulation in the dark tested during and after training (5–195 min) in the same format as before training (0 min). Below the insets, changes in the mean amplitudes of the horizontal (red) and vertical (blue) VOR (learning curves) are plotted. Notably, the amplitude of the horizontal VOR gradually increased, whereas that of the vertical VOR rapidly decreased to almost zero within 30 min, similar to the learning curves during training (Figure 4C). The increase in mean (±SD) amplitude of horizontal eye velocity and the decrease in mean amplitude of vertical eye velocity before and after training are significant (0.3 ± 0.6 vs. 3.4 ± 1.1 °/s, *p* < 0.001 for horizontal eye velocity; 12.5 ± 4.1 vs. 0.7 ± 0.4 °/s, p < 0.001 for vertical eye velocity).

The learning curves indicated that the vertical-dominant VOR responding to translational head motion in the dark became horizontally dominant after training. In naïve goldfish, the vertical VOR is robustly induced by roll-tilt head motion. Since the vertical VOR during translation is suppressed after training, it might also be suppressed during roll-tilt head rotation. This possibility is discussed in the next section.

### 3.4 VOR during roll-tilt before and after training

Figure 5 illustrates changes in the horizontal and vertical VOR eye velocities during roll-tilt stimulation in the dark before and after training (Figure 2D, cyan rectangles 1 and 2) in the same format as in Figures 3, 4.

Both before (A) and after training (B), vertical eye velocity traces were prominently observed (blue, bottom panels), not suppressed, compensatory to roll-tilt angular velocity and GIA angular velocity in the dark (the second panels from the top), while the horizontal eye velocity remained minimal (red, the second panels from the bottom). As illustrated in Figure 5D, the mean eye velocity amplitudes in both horizontal (red) and vertical (blue) VOR were not significantly different before and after training (0.6 ± 0.6 vs. 1.3 ± 2.0 °/s, *p* = 0.157 in horizontal eye velocity; 29.1 ± 5.4 vs. 25.4 ± 4.7 °/s, *p* = 0.060 in vertical eye velocity).

### 3.5 Kalman filter simulations

To interpret these results, we employed a modified version of the Kalman filter model for spatial orientation formation (18). We first validated the model by confirming that it reproduces basic vestibular-visual oculomotor behaviors in goldfish and then applied the model to interpret changes in the VOR and those corresponding to spatial orientation formation.

#### 3.5.1 Model validation

**Figure 7:**
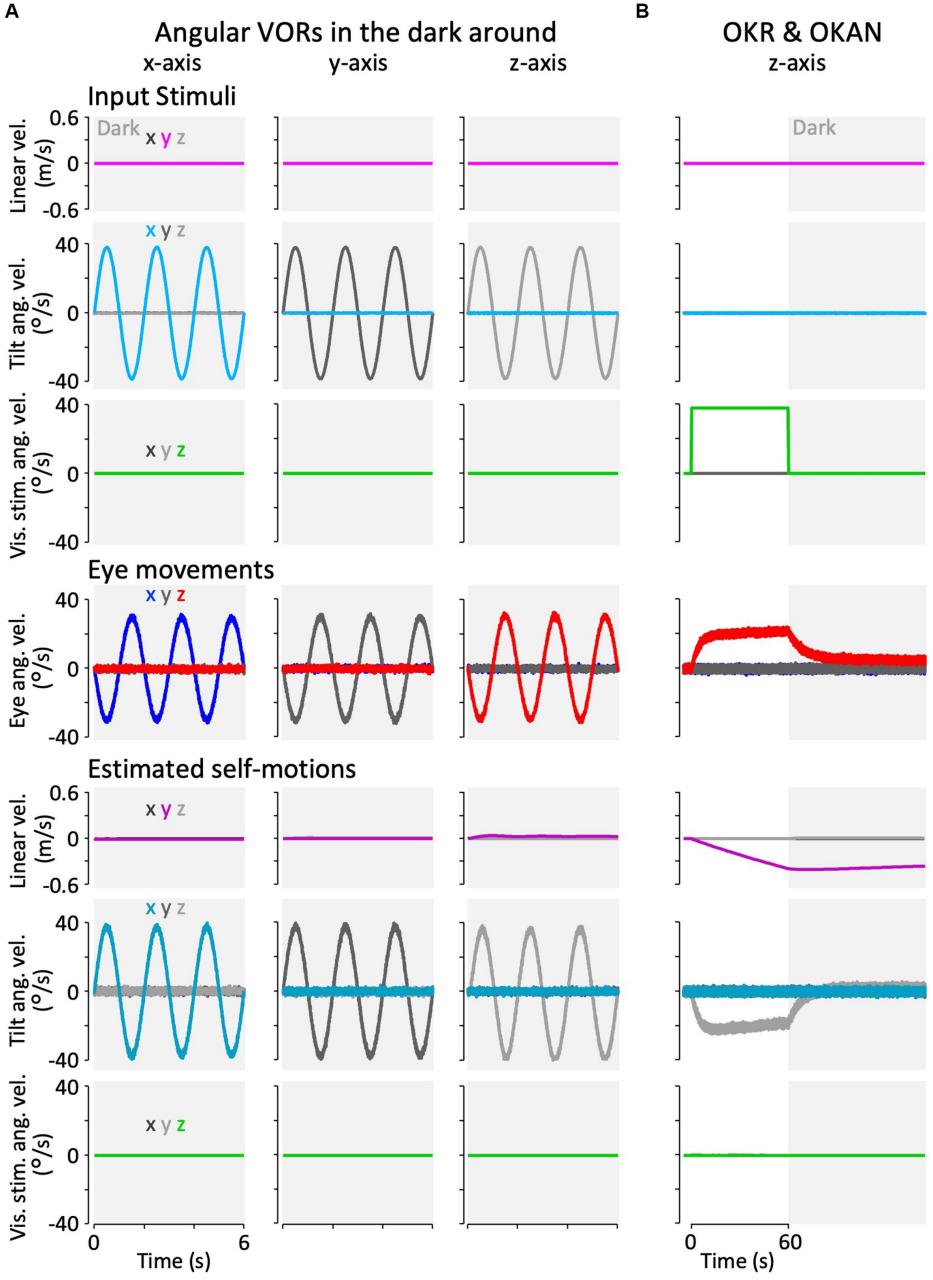
Results of the modified Kalman filter model simulation for model validation. **(A)** Simulation results of angular VORs around the *x*-, *y*-, and *z*-axes. **(B)** Simulation results of OKAN around the z-axis. In panels **(A,B)**, the top three rows represent input stimuli (vestibular or visual), the fourth row from the top depicts eye movements, and the bottom three rows show internally estimated self-motions corresponding to their respective input stimuli. The colors “*x*,” “*y*,” and “*z*” in the inset of the leftmost column indicate the directions (rotation around the *x*-, *y*-, and *z*-axes, or translation along the *x*-, *y*-, and *z*-axes). All traces display the average results of the 12 simulation runs. Traces representing minimal values overlap at the baseline and are not all visible.

Figure 7A shows the model simulation results for the angular VOR in the dark around each rotational axis. Robust compensatory eye velocity was reproduced as reported in previous behavioral experiments (34, 35). The stimuli (linear head, angular head, and angular visual velocities) were estimated as provided. For example, when head rotation was provided in the dark around the *x*-axis (Figure 7A, panel in the first column from the left and second row from the top), eye velocity was produced only around the *x*-axis in the opposite direction to head rotation (Figure 7A, panel in the first column from the left and third row from the top), while head rotation around the *x*-axis was estimated close to the provided head rotation (Figure 7A, panel in the first column from the left and second row from the bottom).

Figure 7B shows the model simulation result of the OKR and OKAN, which were induced by constant velocity visual stimulation around the *z*-axis for 60 s, followed by no stimulation in the dark. It has been demonstrated in goldfish and other species, including primates, that eye velocity gradually increases around the *z*-axis to catch up with the visual stimulus velocity while the stimulation is presented (OKR) and keeps moving in the dark for a while after the stimulation is turned off (OKAN) (36, 37). As in the experimental data, the model reproduced both OKR and OKAN eye velocities (Figure 7B, fourth panel from the top). Notably, head rotation around the *z*-axis was estimated instead of the visual stimulation velocity as the source of the OKR and OKAN eye velocities, as shown in the second panel from the bottom of Figure 7B. In addition, owing to the similarity of visual stimuli caused by rotational and linear head velocity, the slightly accumulated head linear velocity along the *y*-axis was estimated while visual stimulation was provided, and it persisted after OKS was terminated (third panel from the bottom, Figure 7B). All other estimated self-motions from Figures 7A,B were close to zero.

**Figure 8:**
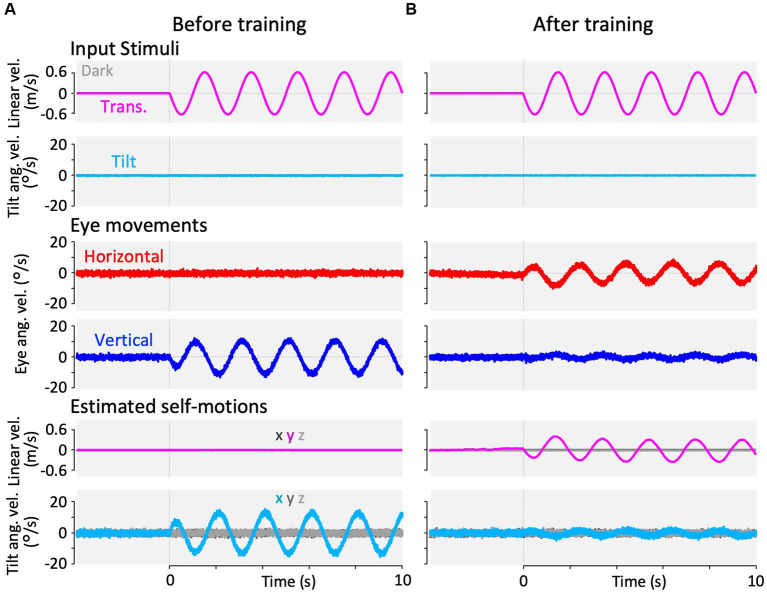
Results of our modified Kalman filter model simulation in response to interaural translational head acceleration in the dark. In the Kalman filter model, the process noise + passive stimuli standard deviation of interaural linear acceleration was set to a small value (0.001 G) at panel **(A)** and a large value (0.180 G) at panel **(B)**, to reproduce the translation test paradigms before and after training. The top two rows show input stimuli, with linear velocity stimulation along the *y*-axis (magenta) and tilt angular velocity around the *x*-axis (cyan). Tilt angular velocity around the *x*-axis is 0 during this stimulation. The third and fourth rows from the top show the simulation results of horizontal eye velocity around the *z*-axis (red) and vertical eye velocity around the *x*-axis (blue). The bottom two rows show the simulation results of linear velocity along the *x*-, *y*-, and *z*-axes (dark gray, dark magenta, and light gray, respectively) and angular velocity around the *x*-, *y*-, and *z*-axes (dark cyan, dark gray, and light gray, respectively), estimated by the Kalman filter. Eye movements and estimated self-motions traces show the average traces of the 12 simulation runs.

**Figure 9:**
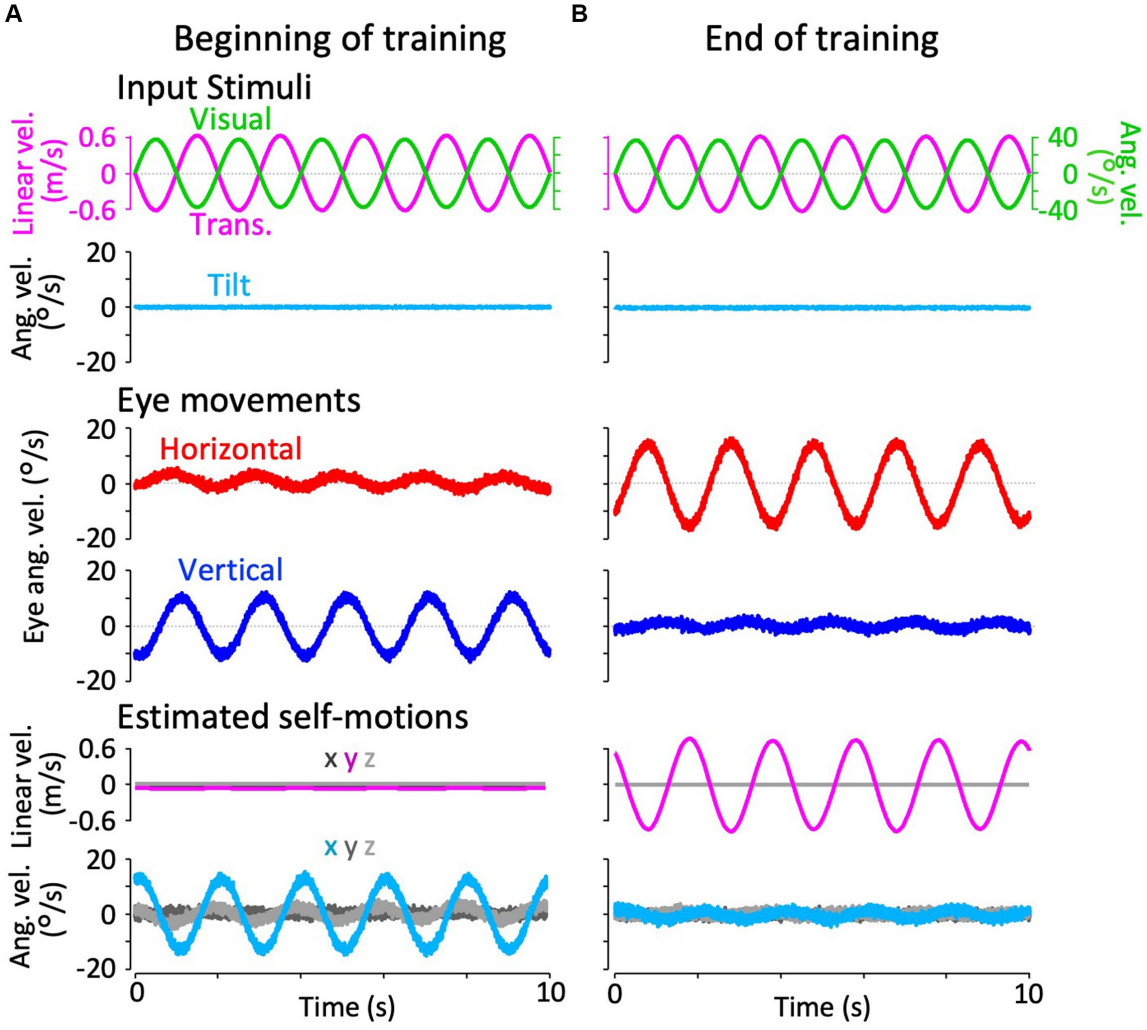
Results of our modified Kalman filter model simulation in response to interaural translational head acceleration along with OKS. In the Kalman filter model, the process noise + passive stimuli standard deviation of interaural linear acceleration was set to a small value (0.001 G) at **(A)** and a large value (0.180 G) at **(B)** to reproduce training paradigms at the beginning and end of the training. The top two rows show input stimuli, with linear velocity stimulation along the *y*-axis (magenta), OKS velocity around the *z*-axis (light green), and tilt angular velocity around the *x*-axis (cyan). Tilt angular velocity is 0 during this stimulation. The third and fourth rows from the top show the simulation results of horizontal eye velocity around the *z*-axis (red) and vertical eye velocity around the *x*-axis (blue). The bottom two rows show the simulation results of linear velocity along the *x*-, *y*-, and *z*-axes (dark gray, dark magenta, and light gray, respectively), and angular velocity around *x*, *y*, and *z*-axis (dark cyan, dark gray, and light gray, respectively), estimated by the Kalman filter. Eye movements and estimated self-motions traces show the average traces of the 12 simulation runs.

**Figure 10:**
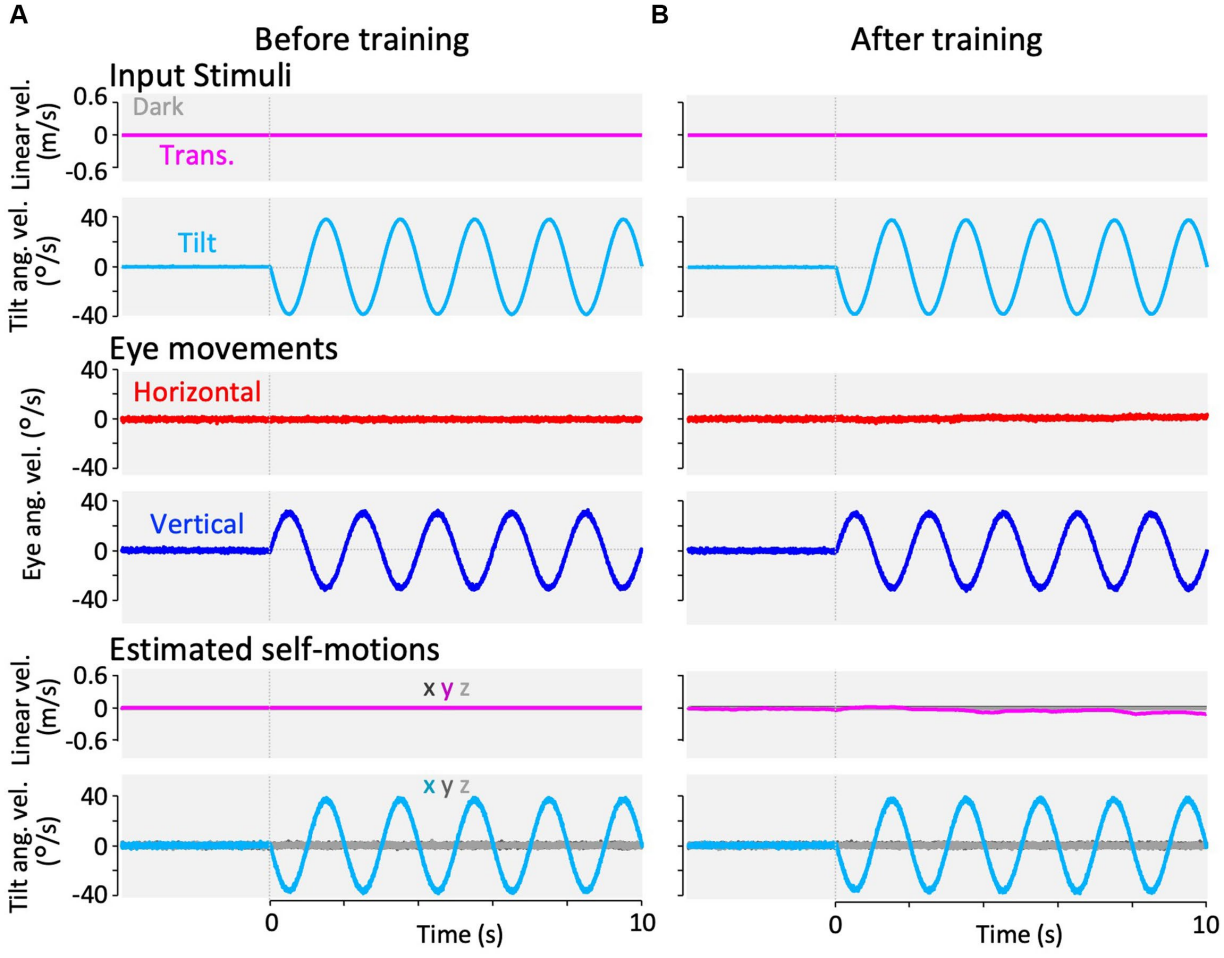
Results of our modified Kalman filter model simulation in response to roll-tilt head rotation in the dark. In the Kalman filter model, the process noise + passive stimuli standard deviation of interaural linear acceleration was set to a small value (0.001 G) at panel **(A)** and a large value (0.180 G) at panel **(B)** to reproduce roll-tilt test paradigms before and after training. The top two rows show input stimuli, with linear velocity stimulation along the *y*-axis (magenta) and tilt angular velocity around the *x*-axis (cyan). Linear velocity stimulation (magenta) is 0 during this stimulation. The third and fourth rows from the top show the simulation results of horizontal eye velocity around the *z*-axis (red) and vertical eye velocity around the *x*-axis (blue). The bottom two rows of panels show the simulation results of linear velocity along the *x*-, *y*-, and *z*-axes (dark gray, dark magenta, and light gray, respectively), and angular velocity around the *x*-, *y*-, and *z*-axes (dark cyan, dark gray, and light gray, respectively), estimated by the Kalman filter. Eye movements and estimated self-motions traces show the averaged traces of 12 simulation runs.

The simulated erroneous estimation of head rotation along with OKR and OKAN also suggests the reproduction of “vection,” an erroneous perception of head rotation in the opposite direction of the visual stimulus (38–40). All lines in Figure 7 were averaged from 12 simulations, similar to the results of the goldfish experiments (Figures 8–10).

#### 3.5.2 Translation and tilt VOR before, during, and after training

Using the same set of model parameters as those used for the model validation, we simulated the VOR in response to translational acceleration and tilt stimulation in the dark, which were used in the current experiment (frequency: 0.5 Hz, GIA amplitude: 0.2 G). Figure 8 shows the simulation results of the translated VOR in the dark before and after training. Before training, the horizontal VOR eye velocity was minimal, as shown in Figure 8A, whereas the vertical VOR was robustly generated, as the current result shown in Figure 3A. Notably, the vertical VOR was caused by a false estimate of the tilt angular velocity around the *x*-axis (fourth panel from the top, Figure 8A), whereas the linear velocity was estimated to be almost zero. To express the animal’s increased experience in translation acceleration, we increased the standard deviation of the linear head acceleration input along the *y*-axis (Table 2, $\sigma_{y}^{A}$ ) from 0.001 G before training to 0.180 G after training while other parameters were unchanged. This manipulation resulted in changes in the VOR after training, as found in the current experiment shown in Figure 3B. Robust horizontal VOR eye velocity was produced, while the vertical VOR was significantly suppressed after training, as illustrated in Figure 8B (after training). Notably, both the linear translation, and tilt angular velocities were correctly estimated after training.

Figure 9 illustrates the simulation results of the VOR with the parameter set for the beginning of training (same as the model validation) and for the end of training in response to visual-translation stimulation. As the current results shown in Figures 4A,B, the minimal horizontal eye velocity before training was enhanced after training (Figure 9, third row from the top), whereas the distinctive vertical eye velocity before training was suppressed significantly after training (Figure 9, fourth row from the top).

Similarly, Figure 10 shows the simulation of VOR with the parameter set before and after training in response to the roll-tilt stimulus in the dark, reproducing the current results (Figures 5A,B). In other words, the horizontal and vertical VORs were unchanged after the training (Figure 10, third and fourth rows from the top). All other estimated self-motions from Figure 8 to Figure 9 were close to zero.

The Kalman gains utilized in this simulation are presented in the Supplementary Results, section 3.5.2. These Kalman gains were computed using the formula provided in the Supplementary Methods, section 2.6.

## 4 Discussion

It has long been argued how the tilt-translation ambiguity arising from peripheral sensory organs (i.e., the otolith) can be resolved to accurately estimate spatial orientation. Notably, humans perceive translational motion in parallel with presenting horizontal VOR during high-frequency (>4 Hz) periodic interaural translations (8, 15, 28, 41), whereas tilt perception and torsional or vertical VOR are induced during low-frequency (<1/20 Hz) or sustained linear accelerations in the dark (4, 8, 10, 11, 28, 42, 43). The VORs in monkeys have also been found to behave similarly (9, 41). In particular, tilt perception during low-frequency or sustained linear acceleration is referred to as “somatogravic illusion,” which has been causing many fatal aviation accidents (4, 5). These phenomena indicate that, in the case of high-frequency periodic linear translation, both humans and monkeys can resolve the ambiguous otolith response and interpret the translation as translation. However, with low-frequency translation or sustained linear acceleration, the ambiguity remains unresolved, resulting in the misinterpretation of translation as a tilt. Two theories have been proposed as the neural mechanisms interpreting the phenomena observed during linear translation.

The first is the frequency-selective segregation theory (9–11). Paige et al. suggested that animals possess high-pass filters for estimating linear translation and low-pass filters for estimating tilt, thus facilitating discrimination between the two types of motion using ambiguous otolith signals. In their theory, the brain filters out high-frequency linear excursions, such as everyday walking, thereby reducing the impact of transient linear motion on tilt detection. Although these low-pass filters also reduce high-frequency otolith-based tilt detection, they are effectively complemented by canal-based tilt-detection methods. A high-pass filter aids in translational estimation to avoid misinterpreting commonly experienced prolonged tilts as translations.

The other theory is referred to as the integration or internal model theory. Over the subsequent decades, multiple theoretical studies have suggested that the brain resolves ambiguity by explicitly solving motion equations to estimate gravity and inertial (translational) motion (12, 44–51). Merfeld unified these theories (12), proposing a comprehensive model integrating the properties of otolith organs and semicircular canals using two distinctive mechanisms: velocity storage mechanism (VSM) and tilt estimator. The tilt estimator mathematically integrates gravity vector changes tracked by resolving Goldstein’s equation of motion (14, 52). Laurens and Angelaki further unified visual motion information and suggested that this integrator functions as a low-pass filter for tilt estimation (2). The high-frequency otolith signal (GIA), which was not utilized in the tilt estimation, was interpreted as linear inertial acceleration during passive motion. This concept aligns with the frequency-selective segregation framework (2).

Both of these major theoretical frameworks are supposed to be appropriate for the natural motion experiences of animals (e.g., linear motion at low frequencies is very rare in nature) (2, 9, 15); Laurens and Angelaki further claimed that animal motion experiences adaptively affect neural mechanisms (2). However, few experimental studies have explored how animals adaptively learn to estimate their self-motion using ambiguous sensory information. To unveil the adaptive mechanism, we hypothesized that animals could adapt their estimation mechanisms based on their accumulated motion experiences and conducted behavioral experiments to test this hypothesis.

Our experiments can be succinctly described as an assessment of the cross-axis VOR adaptation in response to translational vestibular stimuli. Furthermore, this assessment serves as an indicator of adaptive changes in spatial orientation (section 4.3). While only one study of cross-axis VOR adaptation during translation has been previously conducted in monkeys (31), our study using goldfish yielded surprising results that were entirely distinct from those of monkeys, providing novel and crucial insights into the process of adaptive changes in spatial orientation. Below, we provide a detailed discussion of the current results in conjunction with simulations using the modified Kalman filter model.

### 4.1 Adaptive changes in the spatial orientation formed during translation

#### 4.1.1 Translational VOR adaptation in goldfish

Before training, naïve goldfish displayed vertical eye velocity (around the *x*-axis) during interaural translation (along the *y*-axis) in the dark, rather than horizontal VOR (around the *z*-axis), which should be more adequate to stabilize vision if the visual scene is presented in front of the fish (Figure 3, Translation Test before training). The occurrence of VOR around the *x*-axis in naïve goldfish during 0.5 Hz translation is suggested to be a consequence of erroneous tilt estimation (see section 4.3). The gradual increase in the vertical VOR response to translation (along the *y*-axis) over the first 10 s (Figure 3A) appears analogous to the phenomenon observed in the horizontal VOR response to yaw rotation in goldfish. We previously observed this phenomenon in an unreported experiment. It was characterized by the goldfishes’ initial low sensitivity (gain) after having been in darkness and at rest for some time and then exposed to vestibular stimulus, whereafter their sensitivity gradually increased. The cause is unclear; however, a similar phenomenon may have occurred in the vertical VOR response to translation (along the *y*-axis, Figure 3A). However, in the vertical VOR response to tilt (around the *x*-axis, Figure 5A), the gain is large directly following the stimulus onset. Considering the differences in which vestibular organs are stimulated in specific movements—the canal and otolith are stimulated simultaneously during tilt, while only the canal is stimulated during yaw rotation and only the otolith during translation—we postulate that the simultaneous stimulation of the canal and otolith leads to this large gain immediately after the stimulus onset.

At the beginning of training, the goldfish exhibited vertical VORs around the *x*-axis. Although these vertical VORs are based on tilt estimation, they do not contribute to the reduction of retinal slip generated by the presentation of lateral OKS moving parallel to the *y*-axis. Persistent retinal slip might teach goldfish the impropriety of vertical VOR, thereby implying an error in the tilt estimation process, leading to a reduction in vertical VOR. It is important to note that persistent retinal slip alone is insufficient to explain the currently observed rapid suppression of the vertical VOR. The canal prediction error based on the absence of actual tilt rotation, as well as persistent retinal slip, is considered to have contributed to the remarkably rapid suppression (see *Role of “canal prediction error” in rapid translational VOR suppression* in this section). Simultaneously, when goldfish perform translational estimation in response to translational vestibular stimuli and express horizontal VOR, this leads to a reduction in retinal slip and visual stability. The reduction in retinal slip might have kept telling the goldfish that their translational estimation was accurate, which, in turn, could explain the current observation of a gradual increase in translational estimation-based horizontal VOR during training (Figure 4C).

Changes in the eye movements of these goldfish were not transient responses (corrections) to the visual stimuli. Both the VOR with OKS (training paradigm) and the response to translational vestibular stimuli in the dark (translation test paradigm) revealed astonishingly rapid suppression of the vertical VOR around the *x*-axis and a gradual increase in the horizontal VOR around the *z*-axis (Figure 3C). This suggests that the goldfish’s interpretation (motion state estimation) of the translational vestibular stimuli has changed. After 3 h of training, despite a gradual increase in the horizontal vestibulo-ocular reflex (VOR), this gain remained relatively small given the speed of the slider. The limited gain suggests that it is challenging for goldfish to estimate a translation of 0.64 m/s solely from vestibular signals because interaural translation occurs too rarely in goldfish to estimate adequately.

During the roll-tilt tests, both before and after training, the amplitude of the horizontal VOR remained consistently low, whereas the amplitude of the vertical VOR remained substantially high. There were no significant differences before and after the training (Figure 5).

Taken together, it is evident that post-training goldfish VOR can distinguish between interaural translation and roll tilt, even in the dark, suggesting that they no longer misinterpret translation as tilt, but correctly interpret it as translation, while tilt is still properly perceived as tilt.

#### 4.1.2 Role of canal prediction error in rapid translational VOR suppression

The rapid suppression of VOR responses to conflicting OKS observed in goldfish has not been previously documented in both the common angular VOR adaptation (53) and the prior case of translational cross-axis VOR adaptation (31). Typically, angular VOR adaptation results in a faster increase than decrease in learning (53). Furthermore, prior to this study, although only Wei and Angelaki (31) had investigated the cross-axis adaptive plasticity of translational VOR, she did not observe any evident suppression of non-compensatory VOR. She investigated monkeys before and after a 2-h exposure to either vertical or torsional OKS accompanied by interaural translation stimuli (0.5 Hz). Akin to the goldfish horizontal VOR enhancement (around the *z*-axis), the monkeys’ adaptation gradually progressed during OKS exposure (around the *x*- or *y*-axis), enhancing their torsional or vertical VOR (around the *x*- or *y*-axis, respectively). However, despite not compensating for the vertical or torsional OKS, the monkeys’ horizontal VOR remained largely unchanged. Interestingly, it was robustly elicited, even at the moment of exposure to the conflicting torsional OKS around the *x*-axis. Wei’s monkey initially exhibited a VOR around the *z*-axis during interaural translation at 0.5 Hz in contrast to our goldfish, which initially exhibited a VOR around the *x*-axis in response to the same stimulus. Thus, they aimed to adapt the monkeys from the VOR around the *z*-axis to either the VOR around the *x*- or *y*-axis in their study.

The rapidly vanishing vertical VOR around the *x*-axis in goldfish in response to conflicted horizontal OKS and the persistent horizontal VOR around the *z*-axis in monkeys, especially in response to conflicted torsional OKS around the *x*-axis, may be explained by the absence of observed canal signals during translation. In particular, during the actual roll-tilt rotation, both otoliths and canals should be observed by animals; however, during the translational motion-induced roll-tilt illusion, canal signals will not be observed. To avoid the error between the canal signals actually observed during translation and the erroneous canal signals predicted based on the roll-tilt illusion (canal prediction error), both trained goldfish and naïve monkeys might have rapidly discarded the erroneous tilt estimation and converged their estimation toward translation.

There are other questions regarding why naïve goldfish can initially only perform roll-tilt estimation and not translational estimation despite the presence of canal prediction errors, why they become capable of translational estimation through training, and why naïve monkeys are already capable of translational estimation from the beginning. Differences in the probability distribution (i.e., their motion experiences) of passive translation are generally used to explain the distinctions between the high- and low-frequency translational VOR in primates (2, 9, 15), similarly, differences in the animals’ experiences can also explain the differences in translational VOR between goldfish and primates at the same frequency (0.5 Hz). The impact of the probability distributions of passive motion and sensory prediction errors on motion estimation is detailed in section 4.2.

### 4.2 Interpretation based on the Kalman filter model

The Kalman filter, which is a mathematical algorithm developed by Rudolf Kalman in the early 1960s, integrates sensor measurements to provide optimal estimates of directly unobservable states in dynamic systems. In 2017, Laurens and Angelaki introduced the Kalman filter into their previous model (2011) (2) and utilized it as a computational mechanism to estimate the head motion states based on visual and vestibular sensor information (i.e., semicircular canals and otoliths) (18). Their new model, which incorporates a Kalman filter, updates the estimates of head motion states, including linear and rotational motion, by adding corrective values obtained by multiplying the Kalman gain with the sensory prediction error between the observed and predicted sensory signals.

To interpret the results of the current VOR adaptation study, we revised the model to simulate VORs in response to translation and tilted head motion in goldfish, based on the estimates of 3D head motion and visual stimulation (OKS) states. To reproduce the observed changes in the VOR (decrease in the vertical VOR and increase in the horizontal VOR) after training, we varied the parameter representing the variance in linear acceleration along the *y*-axis. This allowed us to replicate the cross-axis VOR adaptation in goldfish presented in this study and investigate adaptive changes in spatial orientation, as depicted in Figures 8–10.

In the Kalman filter model, passive motion is estimated by adding correction values based on sensory prediction errors to the predicted estimates (stationary estimations derived from efference copies) generated by the internal model. In other words, the Kalman filter converges the motion state estimates toward the appropriate values by minimizing the sensory prediction errors. This concept aligns with the idea that both trained goldfish and naive monkeys attempt to avoid canal prediction errors and converge their estimations toward translation (as discussed in section 4.1).

Similarly, the Kalman filter model, as mentioned below, is also capable of describing the impact of differences in the probability distribution (i.e., the difference of motion experiences between the goldfish and monkey) during passive translation. These differences presumably led to distinct estimations, such as roll-tilt estimation in naïve goldfish vs. translational estimation in trained goldfish and naïve monkeys. Within the model, the magnitude of the weighting of the correction values based on sensory prediction errors (Kalman gain) depends on the variance in the probability distribution of the passive motion that the animal anticipates. Animals that do not move voluntarily are generally in a state of rest, although they may occasionally experience passive movements because of being pushed or carried. Therefore, the probability distribution of passive motion is typically centered around zero with a small variance. As animals gain more experience with passive motion, the probability distribution becomes wider and the variance increases. For instance, the daily locomotion and jumping activities of naïve monkeys encompass abundant translational and rotational components along the *x*-, *y*-, and *z*-axes (54). This provided young animals being carried by their parents with rich passive experiences and potentially broadened the probability distribution of each passive motion. Passive motions with relatively wide probability distributions (e.g., roll-tilting in naïve goldfish and high-frequency translational motion in primates) can be largely updated in their motion state estimates through corrections based on sensory prediction errors, even when predicting stationary motion using deficient efference copies of motion commands that are generated only during voluntary motion. However, in passive motion with a small variance in the probability distribution, the magnitude of correction based on sensory prediction errors tends to be small. For instance, it is reasonable to assume that goldfish, which possess a spindle-shaped body mainly designed for forward motion, had significantly less exposure to 0.5 Hz and 0.2 G interaural translation compared to humans and monkeys who commonly experience daily 3D-linear excursions. Furthermore, fish such as goldfish, which live independently from their parents shortly after hatching, can be confidently postulated to have less passive motion, excluding drift, compared to young primates that are carried by their parents. This minimal exposure results in a small variance in the probability distribution, which is expected to result in minimal translational estimation. In the Kalman filter model, passive motion is described as a perturbation affecting the motion state variable (18). This input was treated as process noise because it is unpredictable in the brain (55). The process noise of individuals who encounter a larger than normal range of experienced motion (e.g., a sailor) could be larger (55). In the Kalman filter framework, process noise describes actual motion that is unintended (“passive”) and not motion that is commanded by the brain (“active”) (55). In our model simulations, we specifically manipulated the variance of this process noise (Table 2, $\sigma_{y}^{A}$ ).

Based on the simulation results, the results of the goldfish experiments can be interpreted as follows.

Naive goldfish assume that the variance in the probability distribution of translational movements is small; therefore, they do not update their translational estimates and estimate them to remain small, despite the sensory prediction error from the observed sensory signals. After training, the goldfish perceived the probability distribution of translation as having a large variance; therefore, the weight of the translational correction based on the sensory prediction error increased, and the increase in the estimated translation movements caused the goldfish to move their eyes in the horizontal direction. The increase in translational variance that goldfish assume after training can be attributed to training involving the OKS. Therefore, these results support the possibility that goldfish have a neural mechanism for acquiring optimal spatial orientation by updating the variances of self-motion through visual and vestibular information.

If the goldfish solely responded independently to the otolith and canal reaction, without considering tilt and translation estimation, it would be expected to observe a similar amount of change in VOR responses during tilt after training to those during translation, such as the emergence of enhanced horizontal VOR and reduction of vertical VOR due to the component of the otolith adaptation, even when considering that the canal response remained relatively large during tilt. However, our results did not align with these expectations. This indicated that the 3D VOR system was adapted to differentiate between tilt and translation.

Additionally, given that altering the tilt estimator function affects its frequency filtering (2), adjusting the process noise might similarly influence its frequency filtering and alter the frequency cutoff for the tilt or translation estimation. However, in this study, we could not verify whether the frequency cutoff for tilt or translation estimation was shifted by training, which may have manipulated the variance in the process noise. This is because the 0.8-meter-long slider currently available is unable to measure the goldfish VOR across a sufficient range of frequencies. To address this, we plan to prepare a longer slider to test additional frequencies and evaluate any shift in the frequency cutoff in future studies.

The assumption of small variance in the probability distribution of goldfish translational movements and how it is linked to their living environment can be validated in future studies by equipping fish with an inertial measurement unit attached to their heads. This allows the distribution of motion within their actual living environment to be measured and verifies whether it aligns with our assumption. Concerns about the unit potentially impeding swimming can be alleviated by measuring sufficiently large fish, such as fully grown goldfish or carp, which share ecological similarities with typical-sized goldfish.

The Kalman filter model in this study appears to represent visual- and vestibular-based motion state estimates in the initial pathways formed before conscious motion perception formation within subsequent pathways (see detail in section 4.3). While the simulation results suggest that retinal slip during visual-translation training contributed to updating the variance of the passive motion probability distribution (variance of process noise), the adjustment algorithm responsible for manipulating the parameter is not encompassed within the Kalman filter and will be implemented in our subsequent studies.

As shown in the Supplementary Results, section 3.5.2, Kalman gains vary with the stimulus because this model includes estimated gravity in the calculation of Kalman gains, unlike Laurens’ model, which operates linearly and maintains a state transition matrix with constant Kalman gain values over time. This is because we introduced the $\hat{G}$ from the previous time step, obtained using the Kalman filter, into the Kalman filter gain matrix. This enabled us to describe the calculation of state estimates realistically, addressing the issue posed by Laurens’ model regarding the calculation of state estimates using the real state of G , which the brain cannot inherently perceive. Our objective in this modeling was not to derive a model where the animal’s brain always accurately estimates the external world but to replicate the experimental results and examine how the animal’s brain interprets sensory stimuli to output eye movements. In addition, we consider that the brains of animals, which sometimes have erroneous estimations, may not implement a model that functions accurately at all times.

Moreover, the neural mechanisms and brain regions responsible for this variance adjustment occurring outside the framework of the Kalman filter model remain unknown. One possible brain network involved in this learning process could be located in the cerebellum, which has ample evidence of involvement in processing inertial motion and gravity signals (56). In any case, further investigation of this process is warranted.

### 4.3 VOR as an indicator of motion estimation

Instead of the unobservable motion perception of goldfish, we employed VOR as an indicator of motion estimation. The human torsional VOR around the *x*-axis and the horizontal VOR around the *z*-axis serve as rational eye movements for stabilizing vision in front-eyed animals during roll-tilt and interaural translation. Hence, we interpreted the VORs around the *x*- and *z*-axes as the consequences of roll-tilt and interaural translation estimation, respectively. Although goldfish have relatively more lateralized eye positions than humans, we applied this concept to goldfish by assuming that they are also looking forward. This assumption is supported because the area where fish feed actively is located anteriorly, where their mouths are located, and prior studies (57–59) have demonstrated binocular vision in the frontal visual field of some fish. Specifically, some reports suggest that binocular vision is available even in goldfish and their close relatives, Japanese dace, both of which belong to the Cyprinidae family (60, 61). Any uneasiness regarding the assumption that laterally eyed fish are looking forward is alleviated by two factors. First, the optical axis (the axis passing through the center of curvature of a lens or spherical mirror parallel to the axis of symmetry) does not coincide with the visual axis (the axis or chief ray of the actual pencil of rays that enter the pupil and converge to the fovea, coincident with the direction of gaze), even in humans (62). Another is the observation that similar to primates, the visual axis in some fish (presumed to correspond to the area of high cone density on the retina) is directed forward—in the direction of active feeding behavior (63–69). Moreover, the eyes of goldfish are not completely lateral but slightly oriented forward, so their “optical axis” is not entirely parallel to the *y*-axis but is slightly directed forward. Consequently, even if goldfish do not exhibit binocular vision and their direction of sight coincides with the optical axis, compensatory eye movements for translation along the *y*-axis are likely to be horizontal around the *z*-axis. Furthermore, the induction of horizontal VOR in light with OKS in the forward field of view in this study itself could be considered an example of reflexive eye movement that stabilizes the frontal visual field in goldfish.

The VOR in the dark predominantly reflects head-motion estimations based solely on vestibular information. Similarly, because the VOR also shares pathways with the OKR (70), the VOR in light reflects head motion estimations based on both visual and vestibular information. Furthermore, motion perception is thought to be formed through the integration of multimodal sensory information, such as vestibular, visual, and possibly proprioceptive inputs (71, 72). Considering the hierarchical information processing in the brain, it seems reasonable to assume that high-spatiotemporal-resolution visual and vestibular information are initially used for primary self-motion estimation and swiftly employed in rapid motor control. Subsequently, it is suggested that additional sensory inputs (e.g., proprioception) would be integrated into this initial estimation to form conscious motion perception, which appears to require more time. In fact, the visual field stabilization provided by the VOR operates with a short latency of approximately 10 ms (73), which is significantly faster than the perceptual process (74). Therefore, we considered the VOR, which we adopted as an indicator of motion estimation, to share initial pathways with motion perception and to reflect a more quantitative and rapid vestibular-based motion estimation than motion perception itself.

The foundational premise of our idea is the presence of a shared neural mechanism that potentially contributes to the VOR and motion perception (71, 72). Previous findings have demonstrated that VORs reflect animal perceptions of head motion. For example, it has been demonstrated that humans perceive translational motion in parallel with presenting horizontal VOR during high-frequency (>4 Hz) periodic interaural translations (8, 15, 28, 41), while tilt perception and tortional VOR were induced during low-frequency (<1/20 Hz) or sustained linear accelerations in the dark (4, 8, 10, 11, 28, 42, 43). These VORs compensate for perceived head motion in front-eyed animals, although the torsional VOR does not compensate for actual translational head motion. Similarly, both self-motion perception and VOR amplitude exhibited comparable high-pass filter like characteristics with increasing stimulus frequency from 0.025 to 0.4 Hz during whole body yaw rotation (75, 76). Likewise, the similarity in the time constants of decay of post-rotational motion perception and VOR, both in normal subjects (77, 78) and cerebellar patients (79, 80), has been reported. Another similarity between perception and VOR is the amount of noise as a function of the amplitude. Both dynamics depend on the sensory noise (observation noises of otolith signal $F^{\eta }$ , and semicircular canal signal $V^{\eta }$ in Figure 6) (55, 81). These lines of evidence confirm parallel changes in head motion perception and VOR (82). However, discrepancies between the VOR and motion perception have also been observed in specific situations (8, 28, 43, 82–85). For instance, whereas the VOR and motion perception during interaural translation align at sufficiently high or low frequencies (4, 8, 10, 11, 15, 28, 41–43), they do not align within the intermediate frequency range of approximately 0.1–1 Hz (8, 28, 29). Within this intermediate frequency range, translational acceleration stimulation along the y-axis induces a gradual shift in the VOR axis from torsional to horizontal in primates (28, 29). Conversely, within this range (specifically, 0.15–0.6 Hz), humans perceive linear translation (8, 28). Other studies on tilt and linear acceleration (28, 43, 83, 84) and yaw rotation (82, 85) have also reported discrepancies between the VOR and perceived motion. These dual contradictory phenomena—the concordance and discordance between VOR and motion perception—may be explained by the presence of a vestibular-based common neural mechanism that contributes to both VOR and motion perception, along with subsequent neural mechanisms that exclusively affect motion perception. Indeed, studies involving the GABAergic medication 4-aminopyridine in humans and labyrinthectomy in monkeys have demonstrated significant impairments in the VOR and partial impairments in motion perception (71, 72). These findings indicate that these interventions selectively disrupt the vestibular-based shared neural mechanisms contributing to both the VOR and motion perception, whereas the subsequent neural mechanisms exclusively affecting motion perception remain almost unaffected. This suggests that the coexistence of both shared and unshared pathways (71, 72), also supports our idea that rapid vestibular-based motion estimation is initially output as the VOR and that additional sensory inputs are subsequently integrated, forming motion perception.

## 5 Conclusion

In this study, we first demonstrated dynamic cross-axis changes in translational VORs in goldfish, which indicates that accumulating passive motion experiences enables animals to distinguish between passive translation and tilt, even in the dark, by adaptive changes in the motion state estimation mechanism.

We further validated the adaptive change in the motion state estimation mechanism through a model simulation study employing Laurens and Angelaki’s Kalman filter model. We added the eye motor, retina model, apparent rotation velocity model, and eye controller to their model and extended it to 3D to explain our VOR results. The modified Kalman filter model successfully reproduced our experimental results of goldfish cross-axis VOR adaptation by manipulating the parameter that indicates the accumulation of passive translation experiences and represents the adaptive change in motion state estimation from tilt to translation in response to the input stimuli of interaural translation.

While only one study of cross-axis VOR adaptation during translation has been previously conducted in monkeys, our study using goldfish yielded entirely distinct results from those of monkeys, providing novel and crucial insights into the process of adaptive changes in spatial orientation and eye movements through a Kalman filter model simulation. It potentially provides us with an experimentally verifiable model that enables us to perform an objective, non-invasive, and immediate evaluation of motion state estimation and could potentially offer solutions to critical societal issues such as spatial disorientation, which still has a fatal impact on individuals.

## Data availability statement

The original contributions presented in the study are included in the article/Supplementary material, further inquiries can be directed to the corresponding author.

## Ethics statement

The animal study was approved by the animal welfare committee of Chubu University (Approval ID: 202210010). The study was conducted in accordance with the local legislation and institutional requirements.

## Author contributions

ST: Conceptualization, Data curation, Formal analysis, Investigation, Methodology, Project administration, Software, Writing – original draft, Visualization. YS: Software, Writing – review & editing, Investigation, Methodology, Validation, Visualization. TY: Writing – review & editing, Investigation, Methodology, Resources, Software, Validation, Visualization. YH: Conceptualization, Funding acquisition, Project administration, Supervision, Writing – review & editing, Investigation, Methodology, Software, Validation.
