# Supplementary material for: Learning capabilities to resolve tilt-translation ambiguity in goldfish
Source: Front Neurol. 2024 May 7;15:1304496. doi: 10.3389/fneur.2024.1304496 (PMC11106485; doi:10.3389/fneur.2024.1304496)
Supplement: Supplementary file 1 [file Data_Sheet_1.pdf]

Supplementary Methods

2.3.3 Correction for vertical and horizontal eye positions

2.3.3.1 Correction for vertical eye position

Correction for vertical eye position acquired from a 10° horizontally oblique camera (vertical eye camera) was performed using the following analysis method: The vertical eye position (Supplementary Figure 1, orange cone) determined by LABVIEW software (National Instruments, USA) from the camera with a 10° optical axis is a projection of the actual vertical eye position (Supplementary Figure 1, blue cone) that traces a circular path (Supplementary Figure 1, white circle) onto an ellipse (Supplementary Figure 1, yellow ellipse) in a plane orthogonal to the camera's optical axis. The ellipse had a horizontal (short) axis/vertical (long) axis ratio of $\cos 10°$. The distorted vertical eye position θ (Supplementary Figure 1, orange cone) from the camera can be corrected to the actual vertical eye position θ' (Supplementary Figure 1, blue cone) using the function $\theta' = \tan^{-1}(\tan\theta / \cos 10°)$, where θ is the eye position determined by LABVIEW from the camera image relative to where the null position is set to 0°.

2.3.3.2 Correction for horizontal eye position

The slight tilt of the horizontal eye camera (the camera atop the aquarium) away from the z-axis may project a vertical component of eye movement onto the horizontal eye camera, potentially contaminating the eye position data. When the camera's optical axis tilts by the angle α, on the sagittal plane, the actual vertical eye position (Supplementary Figure 2A, blue cone) on the circular trace (Supplementary Figure 2A, white circle) is projected onto the ellipse (Supplementary Figure 2A, yellow ellipse) in the plane orthogonal to the optical axis (Supplementary Figure 2A, yellow ellipse), resulting in contamination of the horizontal eye position data (Supplementary Figure 2A, orange cone). The ellipse has a vertical (short) or horizontal (long) axis ratio of sinα. The contamination effects are usually minimal but significant when the vertical component largely dominates the horizontal component (e.g., during the Roll-Tilt Test). To correct for this, we estimated the pure contamination θ'' due to the vertical component (Supplementary Figure 2I) using the function $\theta'' = \tan^{-1}(\sin\alpha \times \tan\theta')$ and subtracted it from the horizontal eye position data determined by LABVIEW from the eye camera (Supplementary Figure 2C) to obtain the corrected horizontal component (Supplementary Figure 2K), where θ' is the actual vertical eye position estimated by the aforementioned method and α is the tilt angle of the horizontal eye camera estimated by the following method.

2.3.3.3 Estimation of the tilt angle of the horizontal eye camera

The angle α, representing the tilt angle of the optical axis of the horizontal eye camera, was determined as follows (Supplementary Figure 2B–H). We assumed that only saccades and post-saccadic drift caused horizontal eye movements during the roll-tilt test paradigm before training. To identify saccades and high-frequency noise, a custom-made automatic saccade-detection algorithm was applied to approximate the post-saccade drift by connecting a linear line between the end of the saccade and the beginning of the next saccade. The estimated pure horizontal eye position traces (saccades and post-saccadic drifts) and high-frequency noise (Supplementary Figure 2C) were subtracted from the

horizontal eye position trace determined by LABVIEW using the horizontal eye camera (Supplementary Figure 2D), leaving a residual trace interpreted as pure contamination.

The angle α in the function $\theta'' = \tan^{-1}(\sin\alpha \times \tan\theta')$ was estimated using curve fitting with MATLAB (MathWorks, USA) 'fit' and 'fittype' functions (Supplementary Figure 2H). Here, θ' represents the actual vertical eye position estimated using the previously mentioned method, averaged over each period of the vestibular stimulus to form a one-period array (Supplementary Figure 2F), and θ'' represents the estimated pure contamination trace similarly averaged to form a one-period array (Supplementary Figure 2G). During the averaging process of the contamination trace, data points with a duration of <0.5 s and those deviating by 2 times the median absolute deviation from the median were excluded.

2.5.1 Calculation for the rotational angle of the GIA vector.

The gravito-inertial acceleration (GIA) vector is given as a y, z coordinate, (GIA_y, GIA_Z) or ($\cos(\theta_{GIA} - 90°)$, ($\sin(\theta_{GIA} - 90°)$, where θ_{GIA} is the angle of the GIA vector relative to the negative z-axis, rotating around the goldfish's x-axis. GIA_y was provided by an acceleration sensor. During translational motion (Translation Test and Training), the GIA vector angle can be obtained by solving equation $GIA_z = -1$ (G) and $GIA_y/GIA_x = \tan(\theta_{GIA} - 90°)$, as $\theta_{GIA} = \tan^{-1}(\mathrm{Acc.Sensor\ Value})$ following Lichtenberg (1982) (1) (Supplementary Figure 3A). During roll-tilt rotational motion (Roll-Tilt Test), the GIA vector angle can be obtained by solving equation $GIA_\mathrm{y} = \cos(\theta_\mathrm{GIA} - 90°)$, as $\theta_{GIA} = \sin^{-1}(\mathrm{Acc.Sensor\ Value})$ (Supplementary Figure 3B).

Abbreviations

GIA, gravito-inertial acceleration; Hor., horizontal; Ver., vertical; pos., position; CM, contamination removal.

References

1. Lichtenberg BK, Young LR, Arrott AP. Human ocular counterrolling induced by varying linear accelerations. Exp Brain Res (1982) 48(1):127–36. doi: 10.1007/BF00239580

Supplementary Figure Captions

Supplementary Figure 1. Relationship between the biasedly observed vertical eye position determined by LabVIEW (θ, angle of the orange corn) and actual vertical eye position (θ', angle of the blue corn). The actual vertical eye position and its circular orbit (white circle) were projected onto the observed vertical eye position and its elliptical orbit, which flattened horizontally (yellow ellipse

orthogonal to the oblique vertical eye camera's optical axis, ellipticity is $\cos 10°$). Note that the angles depicted in the graphic are exaggerated compared with the actual camera placement.

Supplementary Figure 2. Removal of contamination in the horizontal eye position observed from the tilted horizontal eye camera. (A) Graphical explanation of the relationship between the contamination (θ'', angle of the orange corn) and actual vertical eye position (θ', angle of the blue corn). Actual vertical eye position and its circular orbit (white circle) were respectively projected onto the contamination and its elliptical orbit that flattened vertically (yellow ellipse orthogonal to the tilted horizontal eye camera's optical axis; ellipticity is $\sin\alpha$, where α is the camera tilt angle). Note that the angles depicted in the graphic are exaggerated compared with the actual camera placement. (B)–(K) Series of sample analyses aimed at eliminating contamination from the observed horizontal eye position. (B) Actual vertical eye position from the vertical eye camera. (C) Observed horizontal eye position using a tilted horizontal eye camera. The contamination was prominent while horizontally stationary and moving slowly (orange lines). (D) Horizontal saccades and eye drift. It was assumed to be a purely horizontal eye position in the roll-tilt test before training. (E) Contamination extracted using the function $(\mathbf{E}) = (\mathbf{C}) - (\mathbf{D})$. (F) Period-averaged traces of (B). (G) Average traces of (E). This indicates contamination. (H) Approximate curve that curve fitted the (G)–(F) plot based on the function $(\mathbf{G}) = \tan^{-1}(\sin\alpha) \times \tan(\mathbf{F})$ and MATLAB 'fit' function. This curve specifies the estimated camera angle α. (I) Estimated contamination, calculated as $(\mathbf{I}) = \tan^{-1}(\sin\alpha) \times \tan(\mathbf{B})$. (J) Average trace of $(\mathbf{J}) = (\mathbf{E}) - (\mathbf{I})$. This indicates the effectiveness of contamination removal. (K) Calculated pure Horizontal Eye position, given by $(\mathbf{K}) = (\mathbf{C}) - (\mathbf{I})$. (B)–(K) Series of examples in which the horizontal eye camera is extremely tilted to emphasize the effect of contamination removal. The tilt angles of the cameras were generally much smaller.

Supplementary Figure 3. Positioning of the GIA vector. The GIA vector is given as a y, z coordinate, (GIA_y, GIA_Z) or ($\cos(\theta_{GIA} - 90°)$, ($\sin(\theta_{GIA} - 90°)$, where θ_{GIA} is the angle of the GIA vector relative to negative z-axis, rotating around the goldfish's x-axis. GIA_y was provided by an acceleration sensor. (A) GIA_z during linear translational motion (Translation Test and Training) is -1 (G); (B) length of GIA vector during roll-tilt rotational motion (Roll-Tilt Test) is 1 (G).

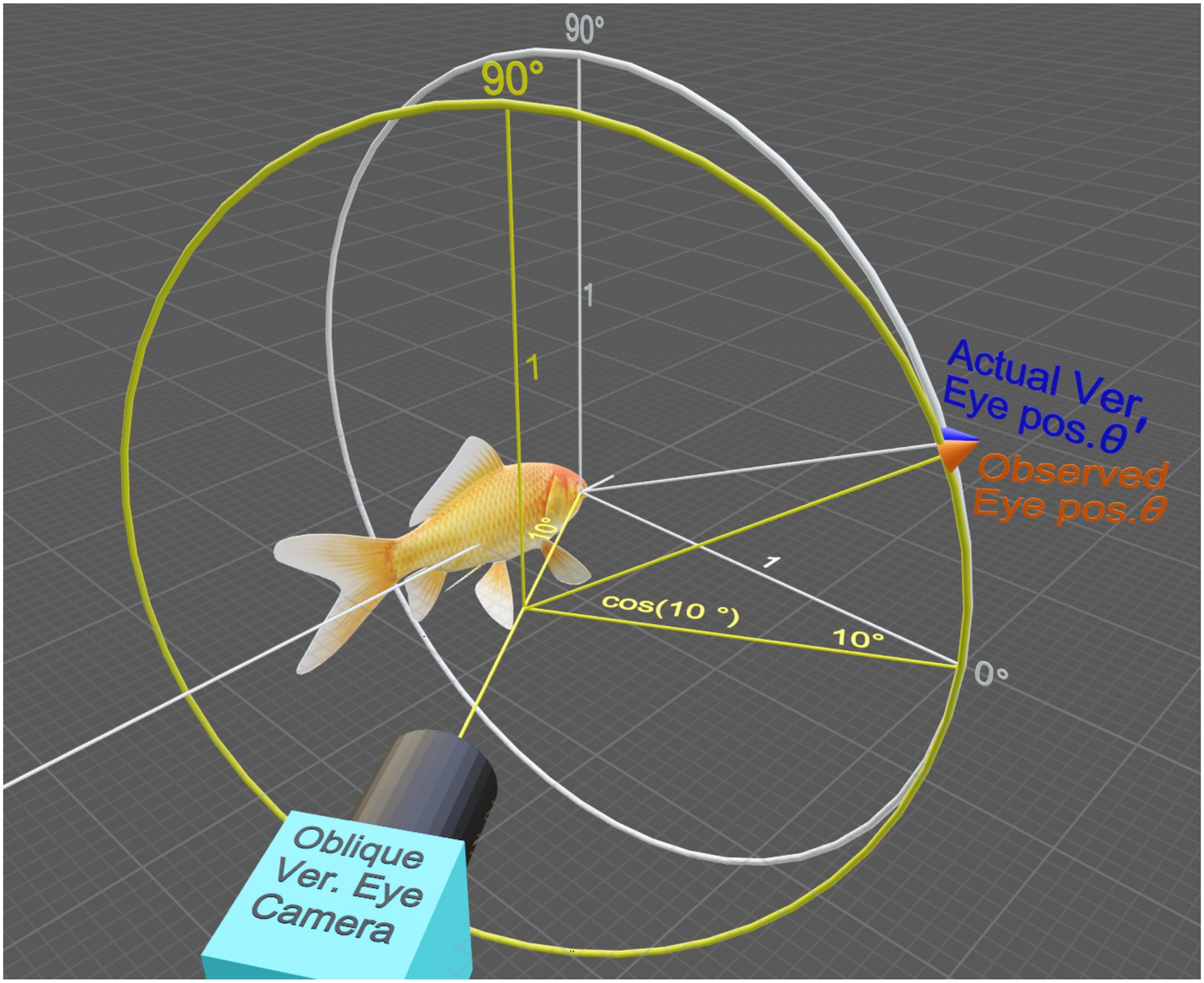

Supplementary Figure 1

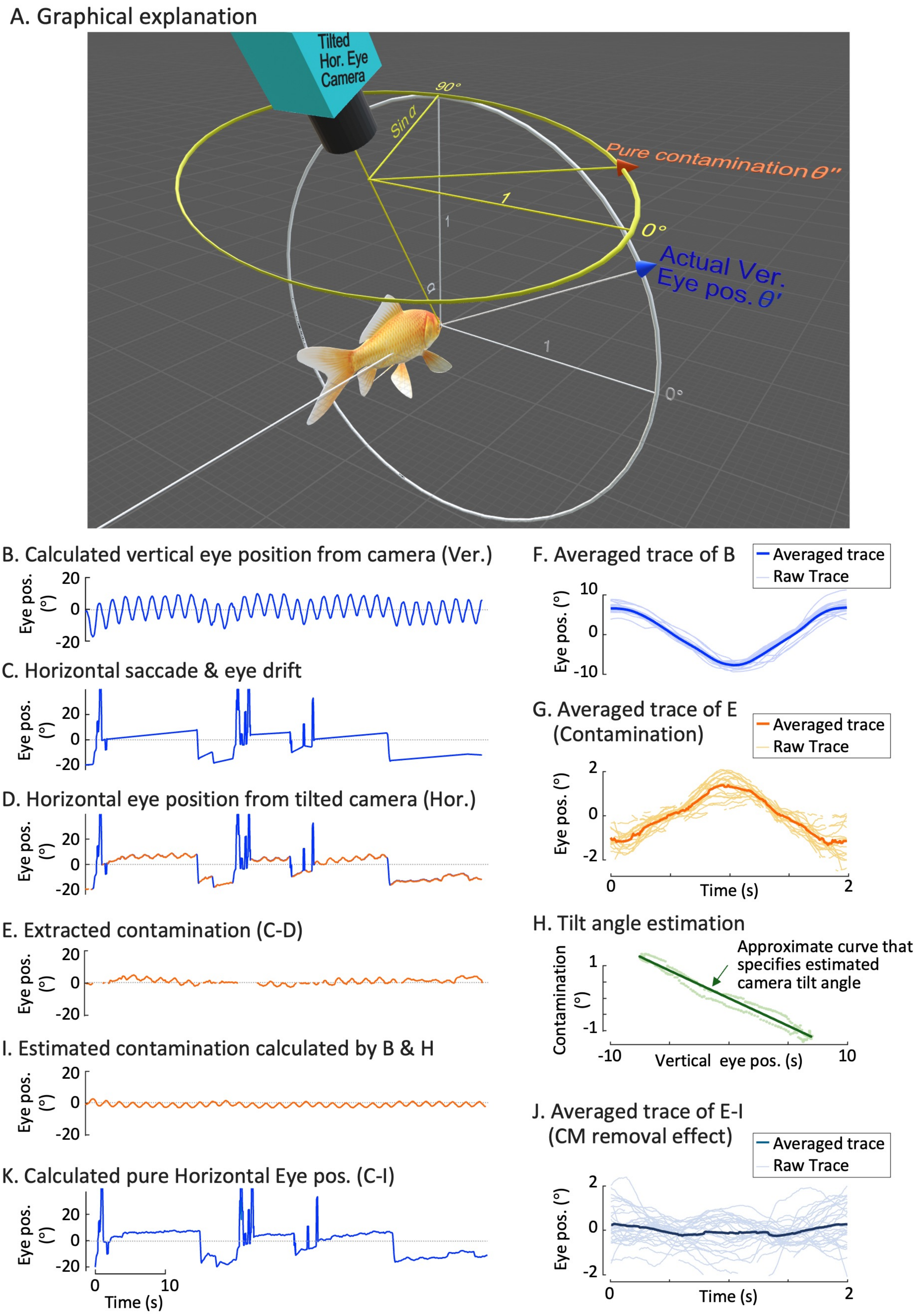

Supplementary Figure 2

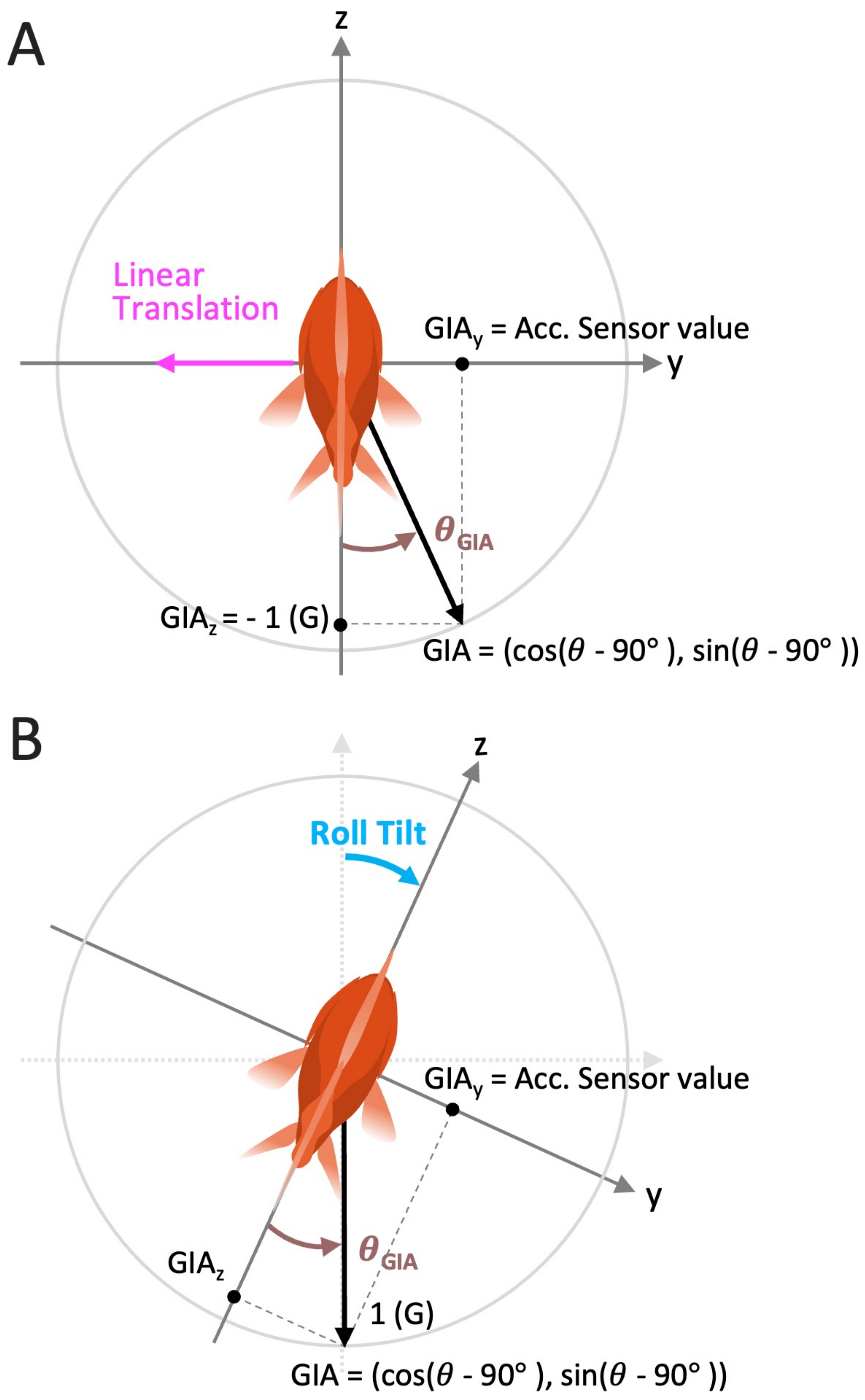

Supplementary Figure 3

2.6 Kalman filter model

2.6.1 Model of head and eye motion with sensors

Model of head motion, eye motion, vestibular sensors and visual sensors

Head rotation velocity

$$\Omega_x(t) = \Omega_x^u(t) + \Omega_x^\varepsilon(t)$$
$$\Omega_y(t) = \Omega_y^u(t) + \Omega_y^\varepsilon(t)$$
$$\Omega_z(t) = \Omega_z^u(t) + \Omega_z^\varepsilon(t)$$

Semicircular Canal endolymph velocity

$$C_x(t) = k_1 C_x(t-\Delta t) + k_2 \Omega_x(t-\Delta t)$$
$$C_y(t) = k_1 C_y(t-\Delta t) + k_2 \Omega_y(t-\Delta t)$$
$$C_z(t) = k_1 C_z(t-\Delta t) + k_2 \Omega_z(t-\Delta t)$$

Gravity

$$G_x(t) = G_x(t-\Delta t) - \Delta t G_z(t-\Delta t)\Omega_y(t-\Delta t) + \Delta t G_y(t-\Delta t)\Omega_z(t-\Delta t)$$
$$G_y(t) = G_y(t-\Delta t) + \Delta t G_z(t-\Delta t)\Omega_x(t-\Delta t) - \Delta t G_x(t-\Delta t)\Omega_z(t-\Delta t)$$
$$G_z(t) = G_z(t-\Delta t) - \Delta t G_y(t-\Delta t)\Omega_x(t-\Delta t) + \Delta t G_x(t-\Delta t)\Omega_y(t-\Delta t)$$

Head liner acceleration

$$A_x(t) = A_x^u(t) + A_x^\varepsilon(t)$$
$$A_y(t) = A_y^u(t) + A_y^\varepsilon(t)$$
$$A_z(t) = A_z^u(t) + A_z^\varepsilon(t)$$

Head linear velocity

$$B_x(t) = B_x(t-\Delta t) + \Delta t A_x(t-\Delta t)$$
$$B_y(t) = B_y(t-\Delta t) + \Delta t A_y(t-\Delta t)$$
$$B_z(t) = B_z(t-\Delta t) + \Delta t A_z(t-\Delta t)$$

Visual velocity

$$\Phi_x(t) = +\Phi_x^\varepsilon(t)$$
$$\Phi_y(t) = +\Phi_y^\varepsilon(t)$$
$$\Phi_z(t) = +\Phi_z^\varepsilon(t)$$

Eye rotation velocity

$$P_x(t) = m_1 P_x(t-\Delta t) + m_2 P_x^u(t-\Delta t) + m_2 P_x^\varepsilon(t)$$
$$P_y(t) = m_1 P_y(t-\Delta t) + m_2 P_y^u(t-\Delta t) + m_2 P_y^\varepsilon(t)$$
$$P_z(t) = m_1 P_z(t-\Delta t) + m_2 P_z^u(t-\Delta t) + m_2 P_z^\varepsilon(t)$$

Semicircular canal signal

$$V_x(t) = \Omega_x(t) - C_x(t) + V_x^\eta(t)$$
$$V_y(t) = \Omega_y(t) - C_y(t) + V_y^\eta(t)$$
$$V_z(t) = \Omega_z(t) - C_z(t) + V_z^\eta(t)$$

Otolith signal

$$F_x(t) = A_x(t) + G_x(t) + F_x^\eta(t)$$
$$F_y(t) = A_y(t) + G_y(t) + F_y^\eta(t)$$
$$F_z(t) = A_z(t) + G_z(t) + F_z^\eta(t)$$

Retina signal

$$\Psi_x(t) = L_i(t)\left(\Phi_x(t) - \Omega_x(t) + rB_y(t) - P_x(t)\right) + \Psi_x^\eta(t)$$
$$\Psi_y(t) = L_i(t)\left(\Phi_y(t) - \Omega_y(t) + rB_z(t) - P_y(t)\right) + \Psi_y^\eta(t)$$
$$\Psi_z(t) = L_i(t)\left(\Phi_z(t) - \Omega_z(t) - rB_y(t) - P_z(t)\right) + \Psi_z^\eta(t)$$

Parameters

Time step
$\Delta t = 0.002$ [s]

Canal dynamics time const
$\tau_C = 0.5$ [s]
$k_1 = 1 - \Delta t/\tau_C$
$k_2 = \Delta t/\tau_C$

Muscle dynamics time const
$\tau_m = 0.003$ [s]
$m_1 = 1 - \Delta t/\tau_m$
$m_2 = \Delta t/\tau_m$

Coefficient for linear stimulus
$r = 4$

Variable to describe light or dark

In the real world model, $L_i(t) = \begin{cases} 1 & \text{if the light is ON} \\ 0 & \text{Otherwise} \end{cases}$

In the Kalman filter model, $L_i(t) = 1$

$L_i(t)$ is always set to 1 in $\boldsymbol{M}$ and $\boldsymbol{T}$ used in the Kalman filter to continue the estimation during the dark interval.

2.6.2 Model in matrix form

State-space equations

$$\boldsymbol{X}(t) = \boldsymbol{D}\,\boldsymbol{X}(t-\Delta t) + \boldsymbol{M}\,\boldsymbol{X}^{u}(t) + \boldsymbol{E}\,\boldsymbol{X}^{\varepsilon}(t)$$

Where:

State variables $\boldsymbol{X}(t) = \left[\Omega_x(t)\ \Omega_y(t)\ \Omega_z(t)\ C_x(t)\ C_y(t)\ C_z(t)\ G_x(t)\ G_y(t)\ G_z(t)\ A_x(t)\ A_y(t)\ A_z(t)\ B_x(t)\ B_y(t)\ B_z(t)\ \Phi_x(t)\ \Phi_y(t)\ \Phi_z(t)\ P_x(t)\ P_y(t)\ P_z(t)\right]'$ The dash denotes a transpose of a matrix.

Input variables $\boldsymbol{X}^{u}(t) = \left[\Omega_x^u(t)\ \Omega_y^u(t)\ \Omega_z^u(t)\ A_x^u(t)\ A_y^u(t)\ A_z^u(t)\ \Phi_x^u(t)\ \Phi_y^u(t)\ \Phi_z^u(t)\ P_x^u(t)\ P_y^u(t)\ P_z^u(t)\right]'$

Stimuli variables + Process noises $\boldsymbol{X}^{\varepsilon}(t) = \left[\Omega_x^\varepsilon(t)\ \Omega_y^\varepsilon(t)\ \Omega_z^\varepsilon(t)\ A_x^\varepsilon(t)\ A_y^\varepsilon(t)\ A_z^\varepsilon(t)\ \Phi_x^\varepsilon(t)\ \Phi_y^\varepsilon(t)\ \Phi_z^\varepsilon(t)\ P_x^\varepsilon(t)\ P_y^\varepsilon(t)\ P_z^\varepsilon(t)\right]'$

$$\boldsymbol{D} = \begin{bmatrix}
0 & 0 & 0 & 0 & 0 & 0 & 0 & 0 & 0 & 0 & 0 & 0 & 0 & 0 & 0 & 0 & 0 & 0 & 0 & 0 & 0\\
0 & 0 & 0 & 0 & 0 & 0 & 0 & 0 & 0 & 0 & 0 & 0 & 0 & 0 & 0 & 0 & 0 & 0 & 0 & 0 & 0\\
0 & 0 & 0 & 0 & 0 & 0 & 0 & 0 & 0 & 0 & 0 & 0 & 0 & 0 & 0 & 0 & 0 & 0 & 0 & 0 & 0\\
k_2 & 0 & 0 & k_1 & 0 & 0 & 0 & 0 & 0 & 0 & 0 & 0 & 0 & 0 & 0 & 0 & 0 & 0 & 0 & 0 & 0\\
0 & k_2 & 0 & 0 & k_1 & 0 & 0 & 0 & 0 & 0 & 0 & 0 & 0 & 0 & 0 & 0 & 0 & 0 & 0 & 0 & 0\\
0 & 0 & k_2 & 0 & 0 & k_1 & 0 & 0 & 0 & 0 & 0 & 0 & 0 & 0 & 0 & 0 & 0 & 0 & 0 & 0 & 0\\
0 & -\Delta t G_z(t) & \Delta t G_y(t) & 0 & 0 & 0 & 1 & 0 & 0 & 0 & 0 & 0 & 0 & 0 & 0 & 0 & 0 & 0 & 0 & 0 & 0\\
\Delta t G_z(t) & 0 & -\Delta t G_x(t) & 0 & 0 & 0 & 0 & 1 & 0 & 0 & 0 & 0 & 0 & 0 & 0 & 0 & 0 & 0 & 0 & 0 & 0\\
-\Delta t G_y(t) & \Delta t G_x(t) & 0 & 0 & 0 & 0 & 0 & 0 & 1 & 0 & 0 & 0 & 0 & 0 & 0 & 0 & 0 & 0 & 0 & 0 & 0\\
0 & 0 & 0 & 0 & 0 & 0 & 0 & 0 & 0 & 0 & 0 & 0 & 0 & 0 & 0 & 0 & 0 & 0 & 0 & 0 & 0\\
0 & 0 & 0 & 0 & 0 & 0 & 0 & 0 & 0 & 0 & 0 & 0 & 0 & 0 & 0 & 0 & 0 & 0 & 0 & 0 & 0\\
0 & 0 & 0 & 0 & 0 & 0 & 0 & 0 & 0 & 0 & 0 & 0 & 0 & 0 & 0 & 0 & 0 & 0 & 0 & 0 & 0\\
0 & 0 & 0 & 0 & 0 & 0 & 0 & 0 & 0 & \Delta t & 0 & 0 & 1 & 0 & 0 & 0 & 0 & 0 & 0 & 0 & 0\\
0 & 0 & 0 & 0 & 0 & 0 & 0 & 0 & 0 & 0 & \Delta t & 0 & 0 & 1 & 0 & 0 & 0 & 0 & 0 & 0 & 0\\
0 & 0 & 0 & 0 & 0 & 0 & 0 & 0 & 0 & 0 & 0 & \Delta t & 0 & 0 & 1 & 0 & 0 & 0 & 0 & 0 & 0\\
0 & 0 & 0 & 0 & 0 & 0 & 0 & 0 & 0 & 0 & 0 & 0 & 0 & 0 & 0 & 0 & 0 & 0 & 0 & 0 & 0\\
0 & 0 & 0 & 0 & 0 & 0 & 0 & 0 & 0 & 0 & 0 & 0 & 0 & 0 & 0 & 0 & 0 & 0 & 0 & 0 & 0\\
0 & 0 & 0 & 0 & 0 & 0 & 0 & 0 & 0 & 0 & 0 & 0 & 0 & 0 & 0 & 0 & 0 & 0 & 0 & 0 & 0\\
0 & 0 & 0 & 0 & 0 & 0 & 0 & 0 & 0 & 0 & 0 & 0 & 0 & 0 & 0 & 0 & 0 & 0 & m_1 & 0 & 0\\
0 & 0 & 0 & 0 & 0 & 0 & 0 & 0 & 0 & 0 & 0 & 0 & 0 & 0 & 0 & 0 & 0 & 0 & 0 & m_1 & 0\\
0 & 0 & 0 & 0 & 0 & 0 & 0 & 0 & 0 & 0 & 0 & 0 & 0 & 0 & 0 & 0 & 0 & 0 & 0 & 0 & m_1
\end{bmatrix}$$

$$\boldsymbol{M} = \begin{bmatrix}
1 & 0 & 0 & 0 & 0 & 0 & 0 & 0 & 0 & 0 & 0 & 0\\
0 & 1 & 0 & 0 & 0 & 0 & 0 & 0 & 0 & 0 & 0 & 0\\
0 & 0 & 1 & 0 & 0 & 0 & 0 & 0 & 0 & 0 & 0 & 0\\
0 & 0 & 0 & 0 & 0 & 0 & 0 & 0 & 0 & 0 & 0 & 0\\
0 & 0 & 0 & 0 & 0 & 0 & 0 & 0 & 0 & 0 & 0 & 0\\
0 & 0 & 0 & 0 & 0 & 0 & 0 & 0 & 0 & 0 & 0 & 0\\
0 & 0 & 0 & 0 & 0 & 0 & 0 & 0 & 0 & 0 & 0 & 0\\
0 & 0 & 0 & 0 & 0 & 0 & 0 & 0 & 0 & 0 & 0 & 0\\
0 & 0 & 0 & 0 & 0 & 0 & 0 & 0 & 0 & 0 & 0 & 0\\
0 & 0 & 0 & 1 & 0 & 0 & 0 & 0 & 0 & 0 & 0 & 0\\
0 & 0 & 0 & 0 & 1 & 0 & 0 & 0 & 0 & 0 & 0 & 0\\
0 & 0 & 0 & 0 & 0 & 1 & 0 & 0 & 0 & 0 & 0 & 0\\
0 & 0 & 0 & 0 & 0 & 0 & 0 & 0 & 0 & 0 & 0 & 0\\
0 & 0 & 0 & 0 & 0 & 0 & 0 & 0 & 0 & 0 & 0 & 0\\
0 & 0 & 0 & 0 & 0 & 0 & 0 & 0 & 0 & 0 & 0 & 0\\
0 & 0 & 0 & 0 & 0 & 0 & 0 & 0 & 0 & 0 & 0 & 0\\
0 & 0 & 0 & 0 & 0 & 0 & 0 & 0 & 0 & 0 & 0 & 0\\
0 & 0 & 0 & 0 & 0 & 0 & 0 & 0 & 0 & 0 & 0 & 0\\
0 & 0 & 0 & 0 & 0 & 0 & 0 & 0 & 0 & m_2 & 0 & 0\\
0 & 0 & 0 & 0 & 0 & 0 & 0 & 0 & 0 & 0 & m_2 & 0\\
0 & 0 & 0 & 0 & 0 & 0 & 0 & 0 & 0 & 0 & 0 & m_2
\end{bmatrix}
\qquad
\boldsymbol{E} = \begin{bmatrix}
1 & 0 & 0 & 0 & 0 & 0 & 0 & 0 & 0 & 0 & 0 & 0\\
0 & 1 & 0 & 0 & 0 & 0 & 0 & 0 & 0 & 0 & 0 & 0\\
0 & 0 & 1 & 0 & 0 & 0 & 0 & 0 & 0 & 0 & 0 & 0\\
0 & 0 & 0 & 0 & 0 & 0 & 0 & 0 & 0 & 0 & 0 & 0\\
0 & 0 & 0 & 0 & 0 & 0 & 0 & 0 & 0 & 0 & 0 & 0\\
0 & 0 & 0 & 0 & 0 & 0 & 0 & 0 & 0 & 0 & 0 & 0\\
0 & 0 & 0 & 0 & 0 & 0 & 0 & 0 & 0 & 0 & 0 & 0\\
0 & 0 & 0 & 0 & 0 & 0 & 0 & 0 & 0 & 0 & 0 & 0\\
0 & 0 & 0 & 0 & 0 & 0 & 0 & 0 & 0 & 0 & 0 & 0\\
0 & 0 & 0 & 1 & 0 & 0 & 0 & 0 & 0 & 0 & 0 & 0\\
0 & 0 & 0 & 0 & 1 & 0 & 0 & 0 & 0 & 0 & 0 & 0\\
0 & 0 & 0 & 0 & 0 & 1 & 0 & 0 & 0 & 0 & 0 & 0\\
0 & 0 & 0 & 0 & 0 & 0 & 0 & 0 & 0 & 0 & 0 & 0\\
0 & 0 & 0 & 0 & 0 & 0 & 0 & 0 & 0 & 0 & 0 & 0\\
0 & 0 & 0 & 0 & 0 & 0 & 0 & 0 & 0 & 0 & 0 & 0\\
0 & 0 & 0 & 0 & 0 & 0 & L_i(t) & 0 & 0 & 0 & 0 & 0\\
0 & 0 & 0 & 0 & 0 & 0 & 0 & L_i(t) & 0 & 0 & 0 & 0\\
0 & 0 & 0 & 0 & 0 & 0 & 0 & 0 & L_i(t) & 0 & 0 & 0\\
0 & 0 & 0 & 0 & 0 & 0 & 0 & 0 & 0 & m_2 & 0 & 0\\
0 & 0 & 0 & 0 & 0 & 0 & 0 & 0 & 0 & 0 & m_2 & 0\\
0 & 0 & 0 & 0 & 0 & 0 & 0 & 0 & 0 & 0 & 0 & m_2
\end{bmatrix}$$

Output equations

$$\boldsymbol{S}(t) = \boldsymbol{T}\,\boldsymbol{X}(t) + \boldsymbol{S}^{\eta}(t)$$

Where:

Sensory variables $\boldsymbol{S}(t) = \left[V_x(t)\ V_y(t)\ V_z(t)\ F_x(t)\ F_y(t)\ F_z(t)\ \Psi_x(t)\ \Psi_y(t)\ \Psi_z(t)\right]'$

Sensory noise $\boldsymbol{S}^{\eta}(t) = \left[V_x^\eta(t)\ V_y^\eta(t)\ V_z^\eta(t)\ F_x^\eta(t)\ F_y^\eta(t)\ F_z^\eta(t)\ \Psi_x^\eta(t)\ \Psi_y^\eta(t)\ \Psi_z^\eta(t)\right]'$

$$\boldsymbol{T} = \begin{bmatrix}
1 & 0 & 0 & -1 & 0 & 0 & 0 & 0 & 0 & 0 & 0 & 0 & 0 & 0 & 0 & 0 & 0 & 0 & 0 & 0 & 0\\
0 & 1 & 0 & 0 & -1 & 0 & 0 & 0 & 0 & 0 & 0 & 0 & 0 & 0 & 0 & 0 & 0 & 0 & 0 & 0 & 0\\
0 & 0 & 1 & 0 & 0 & -1 & 0 & 0 & 0 & 0 & 0 & 0 & 0 & 0 & 0 & 0 & 0 & 0 & 0 & 0 & 0\\
0 & 0 & 0 & 0 & 0 & 0 & 1 & 0 & 0 & 1 & 0 & 0 & 0 & 0 & 0 & 0 & 0 & 0 & 0 & 0 & 0\\
0 & 0 & 0 & 0 & 0 & 0 & 0 & 1 & 0 & 0 & 1 & 0 & 0 & 0 & 0 & 0 & 0 & 0 & 0 & 0 & 0\\
0 & 0 & 0 & 0 & 0 & 0 & 0 & 0 & 1 & 0 & 0 & 1 & 0 & 0 & 0 & 0 & 0 & 0 & 0 & 0 & 0\\
-L_i(t) & 0 & 0 & 0 & 0 & 0 & 0 & 0 & 0 & 0 & 0 & 0 & 0 & 0 & 0 & L_i(t) & 0 & 0 & -L_i(t) & 0 & 0\\
0 & -L_i(t) & 0 & 0 & 0 & 0 & 0 & 0 & 0 & 0 & 0 & 0 & 0 & 0 & rL_i(t) & 0 & L_i(t) & 0 & 0 & -L_i(t) & 0\\
0 & 0 & -L_i(t) & 0 & 0 & 0 & 0 & 0 & 0 & 0 & 0 & 0 & 0 & -rL_i(t) & 0 & 0 & 0 & L_i(t) & 0 & 0 & -L_i(t)
\end{bmatrix}$$

2.6.3 Kalman filter algorithm and control feedback

Optimal estimates

$$\widehat{\boldsymbol{X}}^p(t) = \boldsymbol{D}\,\widehat{\boldsymbol{X}}(t-\Delta t) + \boldsymbol{M}\,\boldsymbol{X}^u(t)$$
$$\widehat{\boldsymbol{S}}^p(t) = \boldsymbol{T}\,\widehat{\boldsymbol{X}}^p(t)$$
$$\boldsymbol{\Delta S}(t) = \boldsymbol{S}(t) - \widehat{\boldsymbol{S}}^p(t)$$
$$\boldsymbol{\Delta}\widehat{\boldsymbol{X}}(t) = \boldsymbol{K}(t)\boldsymbol{\Delta S}(t)$$
$$\widehat{\boldsymbol{X}}(t) = \widehat{\boldsymbol{X}}^p(t) + \boldsymbol{\Delta}\widehat{\boldsymbol{X}}(t)$$

Where:

Predicted state variables $\widehat{\boldsymbol{X}}^p(t)$

Predicted sensor variables $\widehat{\boldsymbol{S}}^p(t) = [\hat{V}_x(t)\ \hat{V}_y(t)\ \hat{V}_z(t)\ \hat{F}_x(t)\ \hat{F}_y(t)\ \hat{F}_z(t)\ \hat{\Psi}_x(t)\ \hat{\Psi}_y(t)\ \hat{\Psi}_z(t)]'$

Feedback variables $\boldsymbol{\Delta}\widehat{\boldsymbol{X}}(t) = [\Delta\hat{\Omega}_x(t)\ \Delta\hat{\Omega}_y(t)\ \Delta\hat{\Omega}_z(t)\ \Delta\hat{C}_x(t)\ \Delta\hat{C}_y(t)\ \Delta\hat{C}_z(t)\ \Delta\hat{G}_x(t)\ \Delta\hat{G}_y(t)\ \Delta\hat{G}_z(t)\ \Delta\hat{A}_x(t)\ \Delta\hat{A}_y(t)\ \Delta\hat{A}_z(t)\ \Delta\hat{B}_x(t)\ \Delta\hat{B}_y(t)\ \Delta\hat{B}_z(t)\ \Delta\hat{\Phi}_x(t)\ \Delta\hat{\Phi}_y(t)\ \Delta\hat{\Phi}_z(t)\ \Delta\hat{P}_x(t)\ \Delta\hat{P}_y(t)\ \Delta\hat{P}_z(t)]'$

Predicted sensor errors $\boldsymbol{\Delta S}(t) = [\Delta\hat{V}_x(t)\ \Delta\hat{V}_y(t)\ \Delta\hat{V}_z(t)\ \Delta\hat{F}_x(t)\ \Delta\hat{F}_y(t)\ \Delta\hat{F}_z(t)\ \Delta\hat{\Psi}_x(t)\ \Delta\hat{\Psi}_y(t)\ \Delta\hat{\Psi}_z(t)]'$

Estimated state variables $\widehat{\boldsymbol{X}}(t) = [\hat{\Omega}_x(t)\ \hat{\Omega}_y(t)\ \hat{\Omega}_z(t)\ \hat{C}_x(t)\ \hat{C}_y(t)\ \hat{C}_z(t)\ \hat{G}_x(t)\ \hat{G}_y(t)\ \hat{G}_z(t)\ \hat{A}_x(t)\ \hat{A}_y(t)\ \hat{A}_z(t)\ \hat{B}_x(t)\ \hat{B}_y(t)\ \hat{B}_z(t)\ \hat{\Phi}_x(t)\ \hat{\Phi}_y(t)\ \hat{\Phi}_z(t)\ \hat{P}_x(t)\ \hat{P}_y(t)\ \hat{P}_z(t)]'$

$\boldsymbol{K}(t)$ is a Kalman gain matrix. $\boldsymbol{D}$, $\boldsymbol{M}$ and $\boldsymbol{T}$ are coefficient matrices. $\boldsymbol{X}^u(t)$ is input variables. $\boldsymbol{S}(t)$ is sensory variables.

Update equation of Kalman gain

$$\boldsymbol{K}(t) = \boldsymbol{L}^p(t)\boldsymbol{T}'(\boldsymbol{T}\boldsymbol{L}^p(t)\boldsymbol{T}' + \boldsymbol{R})^{-1}$$
$$\boldsymbol{L}^p(t) = \boldsymbol{D}\boldsymbol{L}(t-\Delta t)\boldsymbol{D}' + \boldsymbol{Q}$$
$$\boldsymbol{L}(t) = (\boldsymbol{I} - \boldsymbol{K}(t)\boldsymbol{T})\boldsymbol{L}^p(t)$$

$\boldsymbol{I}$ is an identity matrix. $\boldsymbol{L}^p(t)$ and $\boldsymbol{L}(t)$ are covariances of the predicted and updated estimate. $\boldsymbol{Q}$ and $\boldsymbol{R}$ are the covariance matrices of $\boldsymbol{E}\,\boldsymbol{X}^{\varepsilon}$ and $\boldsymbol{S}^{\eta}$.

$$\boldsymbol{Q} = \boldsymbol{E}\begin{bmatrix} (\sigma^{\Omega})^2 & 0 & 0 & 0 & 0 & 0 & 0 & 0 & 0 & 0 & 0 & 0 \\ 0 & (\sigma^{\Omega})^2 & 0 & 0 & 0 & 0 & 0 & 0 & 0 & 0 & 0 & 0 \\ 0 & 0 & (\sigma^{\Omega})^2 & 0 & 0 & 0 & 0 & 0 & 0 & 0 & 0 & 0 \\ 0 & 0 & 0 & (\sigma_x^{A})^2 & 0 & 0 & 0 & 0 & 0 & 0 & 0 & 0 \\ 0 & 0 & 0 & 0 & (\sigma_y^{A})^2 & 0 & 0 & 0 & 0 & 0 & 0 & 0 \\ 0 & 0 & 0 & 0 & 0 & (\sigma_z^{A})^2 & 0 & 0 & 0 & 0 & 0 & 0 \\ 0 & 0 & 0 & 0 & 0 & 0 & (\sigma^{\Phi})^2 & 0 & 0 & 0 & 0 & 0 \\ 0 & 0 & 0 & 0 & 0 & 0 & 0 & (\sigma^{\Phi})^2 & 0 & 0 & 0 & 0 \\ 0 & 0 & 0 & 0 & 0 & 0 & 0 & 0 & (\sigma^{\Phi})^2 & 0 & 0 & 0 \\ 0 & 0 & 0 & 0 & 0 & 0 & 0 & 0 & 0 & (\sigma^{P})^2 & 0 & 0 \\ 0 & 0 & 0 & 0 & 0 & 0 & 0 & 0 & 0 & 0 & (\sigma^{P})^2 & 0 \\ 0 & 0 & 0 & 0 & 0 & 0 & 0 & 0 & 0 & 0 & 0 & (\sigma^{P})^2 \end{bmatrix}\boldsymbol{E}'$$

$$\boldsymbol{R} = \begin{bmatrix} (\sigma^{V})^2 & 0 & 0 & 0 & 0 & 0 & 0 & 0 & 0 \\ 0 & (\sigma^{V})^2 & 0 & 0 & 0 & 0 & 0 & 0 & 0 \\ 0 & 0 & (\sigma^{V})^2 & 0 & 0 & 0 & 0 & 0 & 0 \\ 0 & 0 & 0 & (\sigma^{F})^2 & 0 & 0 & 0 & 0 & 0 \\ 0 & 0 & 0 & 0 & (\sigma^{F})^2 & 0 & 0 & 0 & 0 \\ 0 & 0 & 0 & 0 & 0 & (\sigma^{F})^2 & 0 & 0 & 0 \\ 0 & 0 & 0 & 0 & 0 & 0 & (\sigma^{\Psi})^2 & 0 & 0 \\ 0 & 0 & 0 & 0 & 0 & 0 & 0 & (\sigma^{\Psi})^2 & 0 \\ 0 & 0 & 0 & 0 & 0 & 0 & 0 & 0 & (\sigma^{\Psi})^2 \end{bmatrix}$$

Control feedback

Eye motor command

$$P_x^u(t) = h\,(\hat{\Phi}_x(t) - \hat{\Omega}_x(t))$$
$$P_y^u(t) = h\,(\hat{\Phi}_y(t) - \hat{\Omega}_y(t) + r\hat{B}_z(t))$$
$$P_z^u(t) = h\,(\hat{\Phi}_z(t) - \hat{\Omega}_z(t) - r\hat{B}_y(t))$$

Where: control gain $h = 0.8$
