# Supplementary material for: Learning capabilities to resolve tilt-translation ambiguity in goldfish
Source: Front Neurol. 2024 May 7;15:1304496. doi: 10.3389/fneur.2024.1304496 (PMC11106485; doi:10.3389/fneur.2024.1304496)

Supplementary Results

3.5.2.1 Kalman gains in linear VOR dark before training

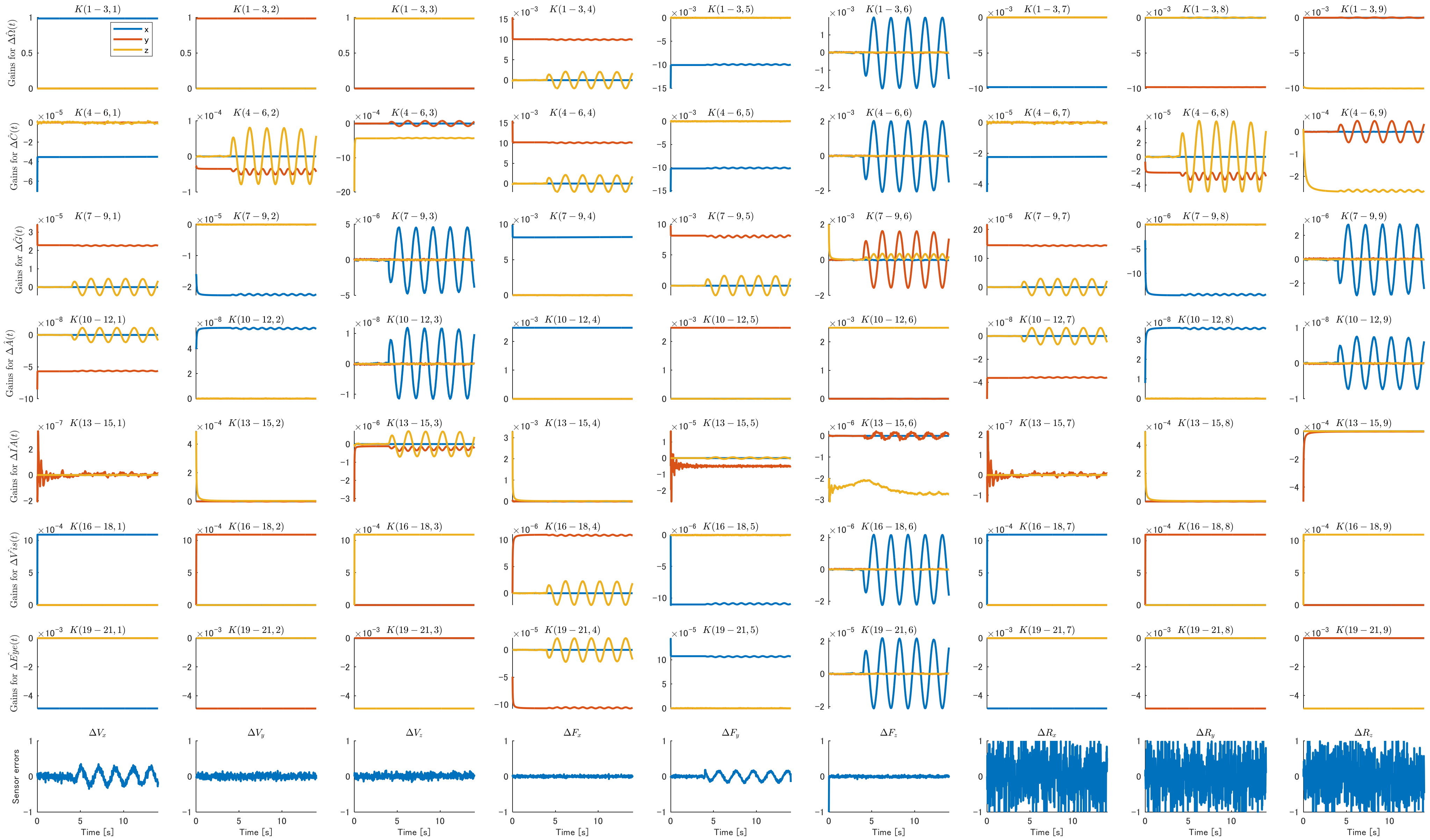

Example:

$$\Delta\hat{\Omega}_x = K(1,1)\Delta V_x + K(1,2)\Delta V_y + K(1,3)\Delta V_z + K(1,4)\Delta F_x + K(1,5)\Delta F_y + K(1,6)\Delta F_z + K(1,7)\Delta R_x + K(1,8)\Delta R_x + K(1,9)\Delta R_z$$

$$\Delta\hat{\Omega}_y = K(2,1)\Delta V_x + K(2,2)\Delta V_y + K(2,3)\Delta V_z + K(2,4)\Delta F_x + K(2,5)\Delta F_y + K(2,6)\Delta F_z + K(2,7)\Delta R_x + K(2,8)\Delta R_x + K(2,9)\Delta R_z$$

$$\Delta\hat{\Omega}_z = K(3,1)\Delta V_x + K(3,2)\Delta V_y + K(3,3)\Delta V_z + K(3,4)\Delta F_x + K(3,5)\Delta F_y + K(3,6)\Delta F_z + K(3,7)\Delta R_x + K(3,8)\Delta R_x + K(3,9)\Delta R_z$$

3.5.2.2 Kalman gains in linear VOR dark after training

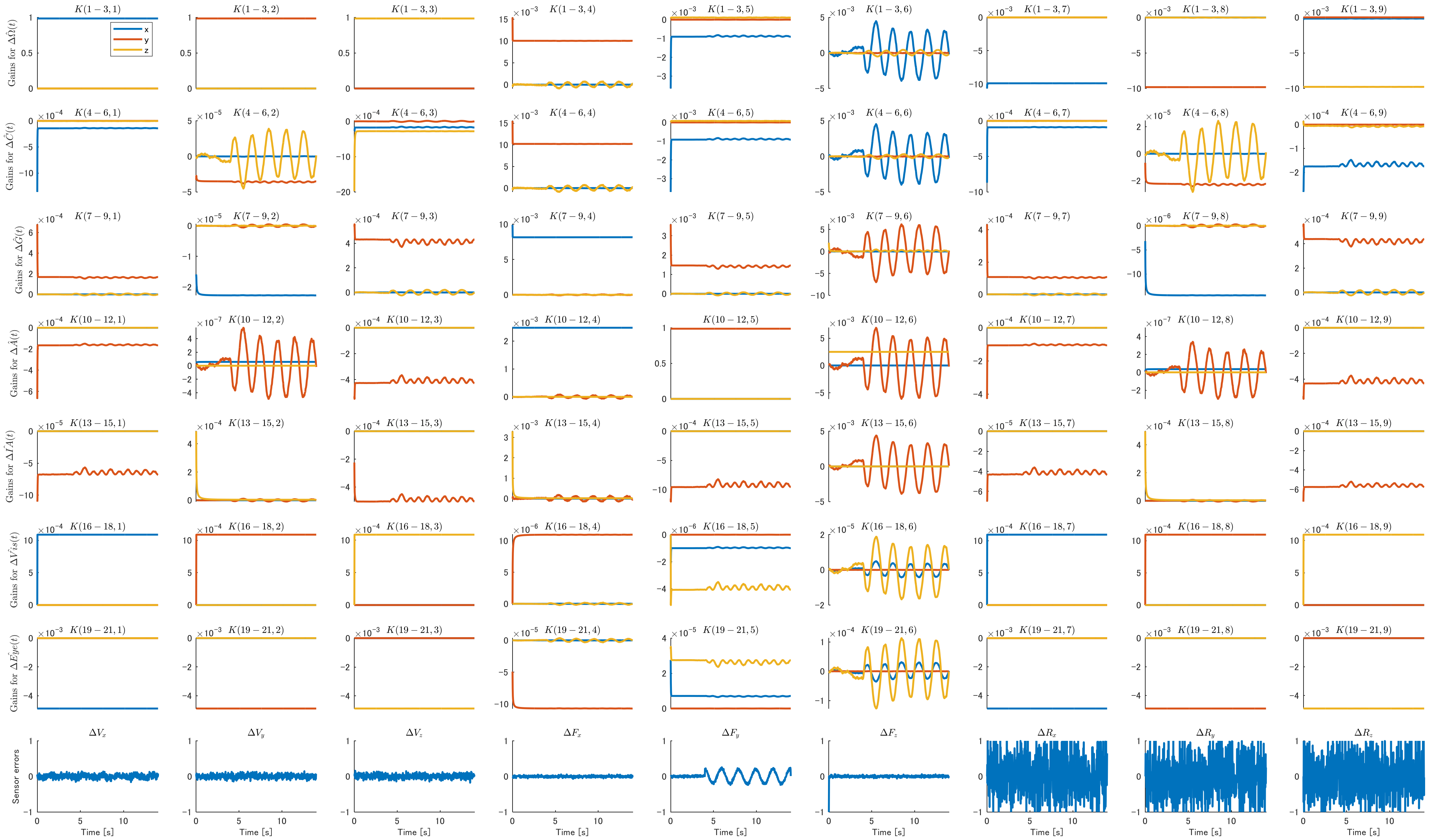

3.5.2.3 Kalman gains in rotate VOR dark before training

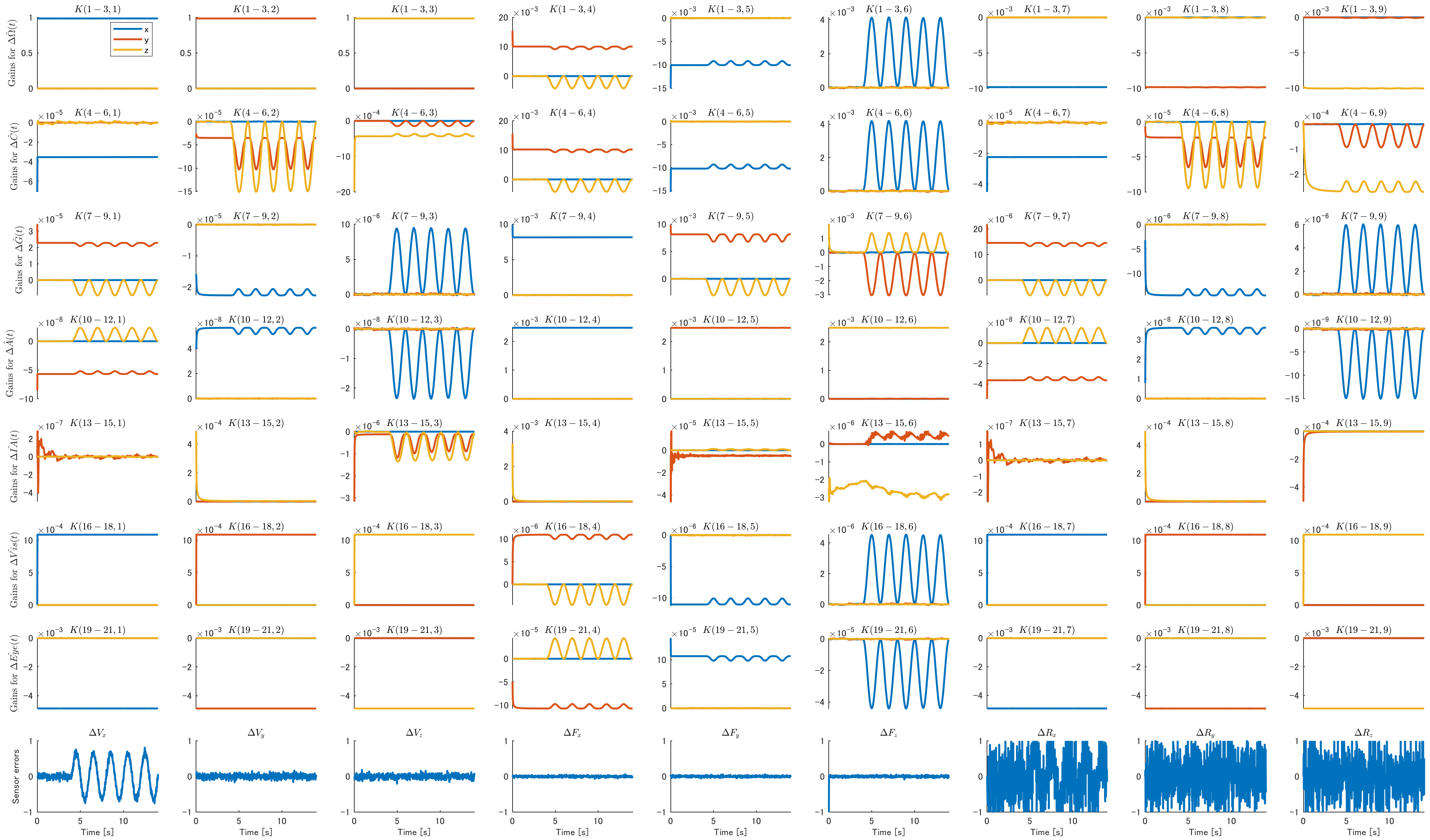

3.5.2.4 Kalman gains in rotate VOR dark after training

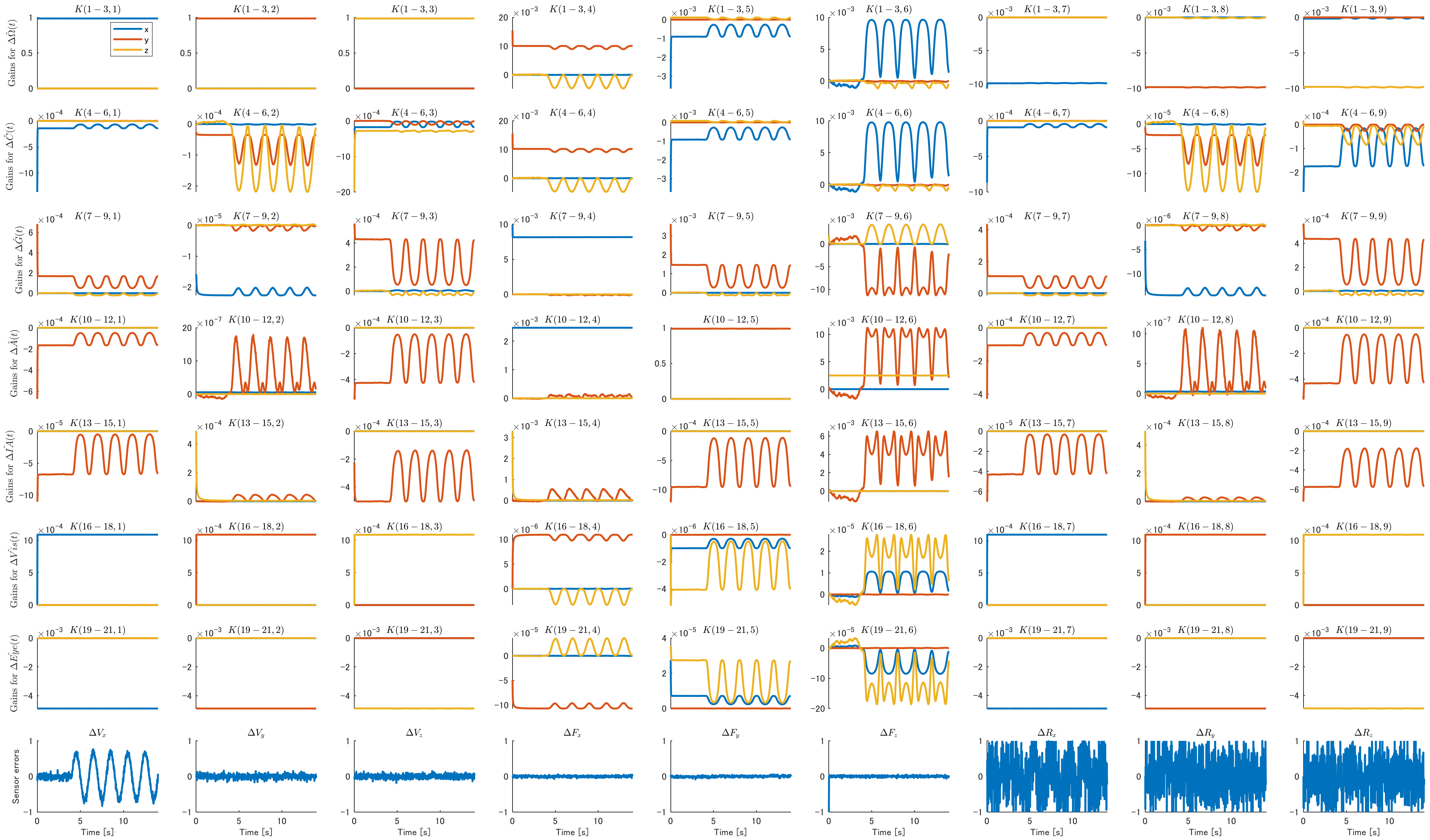

3.5.2.5 Kalman gains in linear VOR light beginning of training

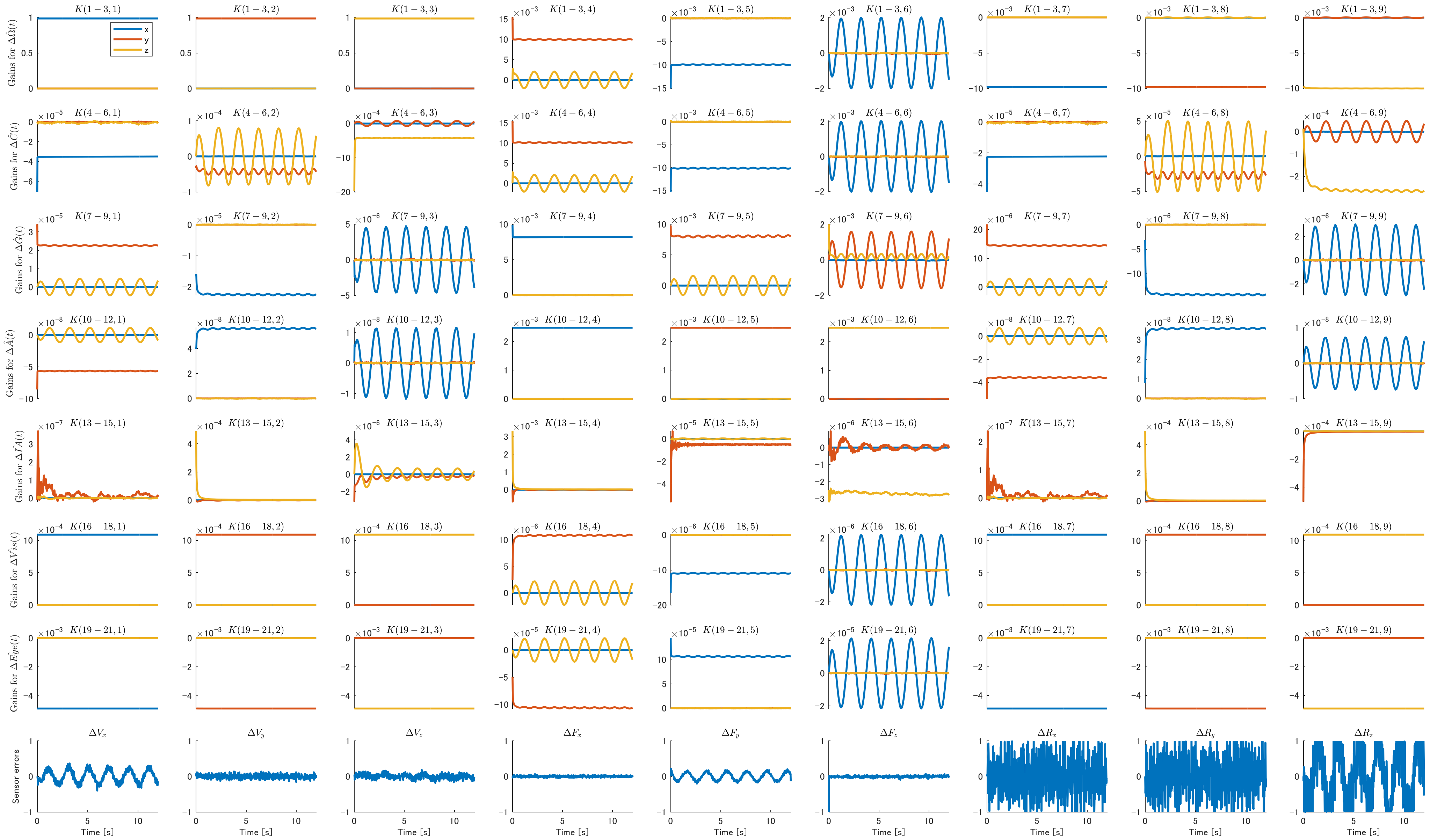

3.5.2.6 Kalman gains in linear VOR light end of training

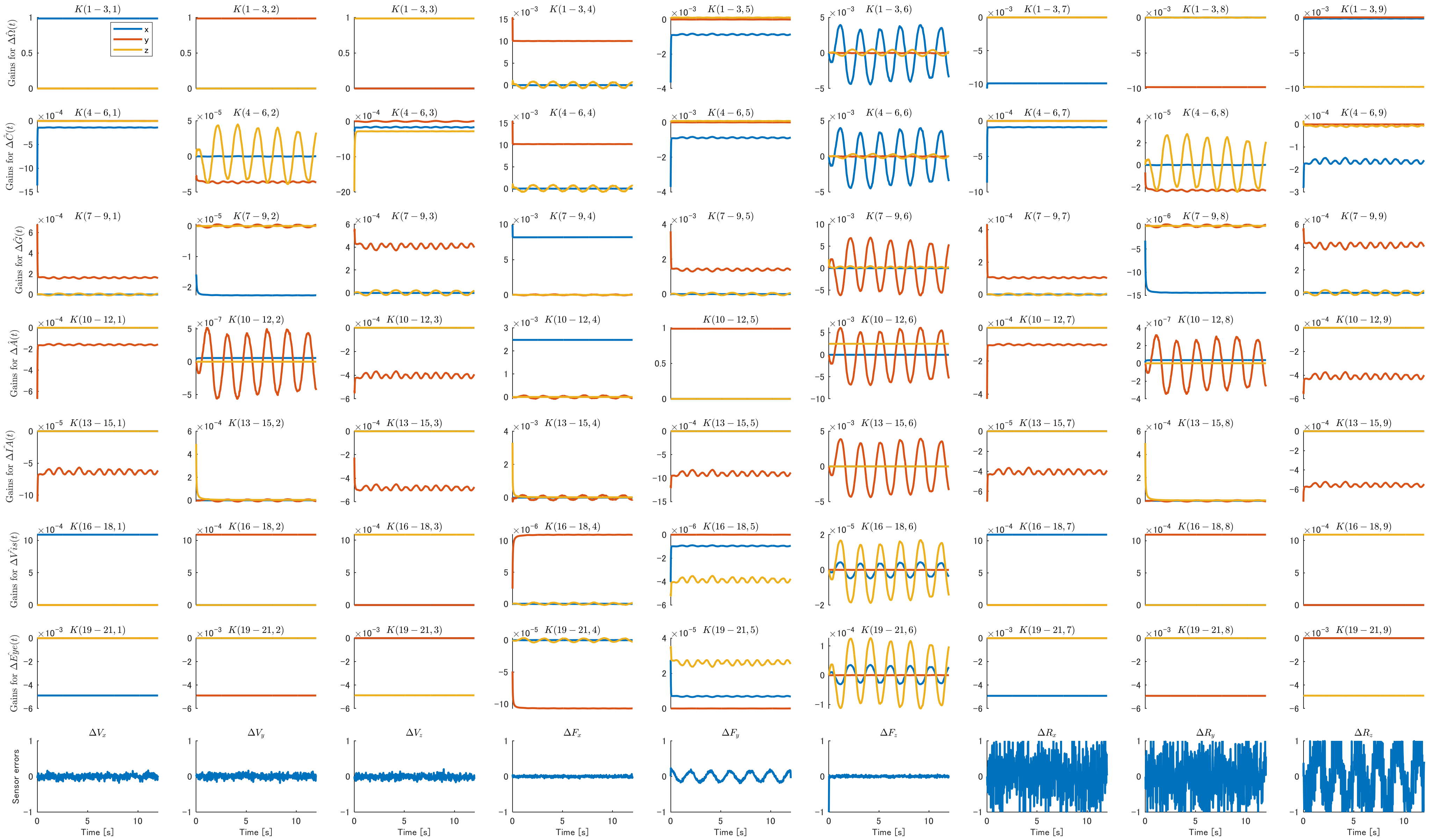

Supplement: Supplementary file 2 [file Data_Sheet_2.pdf]
